# phasebook: haplotype-aware de novo assembly of diploid genomes from long reads

**DOI:** 10.1186/s13059-021-02512-x

**Published:** 2021-10-27

**Authors:** Xiao Luo, Xiongbin Kang, Alexander Schönhuth

**Affiliations:** 1 Life Science & Health, Centrum Wiskunde & Informatica, Amsterdam, The Netherlands; 2 Genome Data Science, Faculty of Technology, Bielefeld University, Bielefeld, Germany

**Keywords:** Genome assembly, Haplotype, Diploid, Long reads

## Abstract

Haplotype-aware diploid genome assembly is crucial in genomics, precision medicine, and many other disciplines. Long-read sequencing technologies have greatly improved genome assembly. However, current long-read assemblers are either reference based, so introduce biases, or fail to capture the haplotype diversity of diploid genomes. We present phasebook, a de novo approach for reconstructing the haplotypes of diploid genomes from long reads. phasebook outperforms other approaches in terms of haplotype coverage by large margins, in addition to achieving competitive performance in terms of assembly errors and assembly contiguity.

**Supplementary Information:**

The online version contains supplementary material available at (10.1186/s13059-021-02512-x).

## Background

There are generally multiple copies of hereditary material in most eukaryotic organisms, where each copy is inherited from one of the ancestors. The copy-specific nucleic acid sequences are called *haplotypes*, and the haplotype-specific contigs that assembly programs compute for reconstructing them are often referred to as *haplotigs*. Most vertebrates (such as human and mouse) and many higher plants (such as maize and arabidopsis) are diploid, which means that there are two copies for each chromosome.

Haplotype reconstruction plays a crucial role in various disciplines. For example, haplotype information is important in functional genomics since there is widespread allele-specific gene expression across the human genome [1]; haplotype information also crucially supports studies on population demography, gene flow, and selection in conservation genomics [2]; the haplotype-specific combinations of genetic variants usually affect disease phenotypes and clinical responses, which is of great concern in precision medicine [3, 4].

Over the last few years, long-read sequencing technologies such as single-molecule real-time (SMRT) sequencing of Pacific Biosciences (PacBio) and nanopore sequencing of Oxford Nanopore Technologies (ONT) have improved genome assembly greatly because sequencing reads are sufficiently long (generally ranging from several kbp to hundreds of kbp, or even to a few Mbp) to also span more complex repeat regions [5–8].

Although long reads have tremendous advantages in terms of read length in comparison with next-generation sequencing (NGS)–derived short reads, they suffer from (substantially) elevated sequencing error rates. In particular, PacBio CLR and ONT reads, as the most representative examples of long reads, have a sequencing error rate as high as 5 to 15%. While PacBio HiFi reads have much lower error rates (< 1*%*), and in comparison with NGS reads are still long, such reads considerably sacrifice on read length in comparison with PacBio CLR and ONT. The significant differences in terms of read length and error characteristics explain why short-read genome assembly tools cannot be directly applied for long-read data; novel approaches are required.

Existing methods for haplotype reconstruction of diploid genome from long reads basically fall into two classes. The first one involves alignment-based methods, which are referred to as *haplotype assembly* programs. Recent works such as WhatsHap [9], HapCut2 [10], and HapCol [11] are designed for diploid haplotype assembly. It is characteristic for these tools to make use of a high-quality reference as a backbone sequence. They align reads to it to call variants using external tools. Subsequently, variants are separated into different haplotypes based on their own phasing algorithms. The output haplotigs of these tools then reflect modified reference sequence patches. However, because only minor modifications of the backbone sequence can be applied, the haplotigs can be affected by non-negligible biases.

For avoiding reference induced biases, de novo assembly-based methods can be used as an alternative. A series of remarkable de novo genome assemblers specialized for long-read sequencing data have been developed so far. FALCON [12], which follows the hierarchical genome assembly process, and its haplotype-aware version FALCON-Unzip are mainly designed for PacBio long reads. Improved Phased Assembler (IPA) [13] is designed to produce phased genome assemblies from PacBio HiFi reads. Canu [14] uses adaptive overlapping algorithm and sparse assembly graph construction which helps to separate repeats and haplotypes, and its modification version HiCanu [15] is tailored for haplotype aware assembly from PacBio HiFi reads. Moreover, Flye [16] tries to generate optimal assemblies using repeat graphs. Generic long-read assemblers Shasta [17] and Wtdbg2 [18] considerably speed up the large-scale long-read assembly based on novel graph representations. Notably, Hifiasm [19] is a haplotype-resolved assembler specifically designed for PacBio HiFi reads. A graph-based approach to haplotype aware diploid assembly was proposed in [20], which combines accurate short-read data and long-read (PacBio) data in a hybrid (employing both NGS and long reads) type framework.

There are various other long-read assembly approaches, all of which are not designed to produce haplotype-aware assemblies. They usually collapse homologous sequences into one consensus sequence and therefore do not distinguish between the haplotypes of a diploid genome sufficiently accurately. We refer the reader to [17, 21] for a comprehensive overview of such tools.

In summary, long-read assemblers are either designed to work for only specific types of data, such as PacBio HiFi or ONT, or they do not address haplotype-aware assembly of diploid genomes by design. In somewhat more detail, there is no method that combines the following three important points: it generates high-quality haplotype-aware genome assemblies only based on long-read data (1); is capable of handling all three of PacBio CLR, PacBio HiFi, and Nanopore reads (2); and does not depend on high-quality reference sequence as a backbone (3).

Here, we present *phasebook* and suggest it as an approach that to the best of our knowledge is the first one to address all (1), (2), and (3) in combination. By bridging the gap in the body of existing work, phasebook appears to be the first approach that presents a framework that allows to reconstruct the haplotypes of diploid genomes de novo from long reads without having to specialize in a particular sequencing technology.

We evaluate phasebook on different long-read sequencing data, namely PacBio CLR/HiFi reads and Nanopore reads. Benchmarking results on both simulated and real data indicate that our method outperforms state-of-the-art tools (both haplotype assembly and de novo assembly approaches) in terms of various aspects. Thereby, an application scenario of major interest is to reconstruct individual haplotypes of the major histocompatibility complex (MHC) region, spanning ∼ 5 Mbp of chromosome 6 and playing a pivotal role in the adaptive immune system. Because of its great hereditary variability, haplotype identity is of utmost interest with respect to numerous human diseases and the corresponding medical treatments. Overall, phasebook generates more complete, more accurate, and, with respect to full contig length statistics, comparably long haplotigs compared with other approaches in this particularly challenging case.

## Results

We have designed and implemented phasebook, a novel approach to assemble individual haplotypes of diploid genomes from long-read sequencing data, namely PacBio HiFi, PacBio CLR, and Oxford Nanopore reads. In this section, we provide a high-level description of the workflow and evaluate its performance on both simulated and real data, in comparison with an exhaustive selection of existing state-of-the-art tools. These tools include both de novo long-read assemblers and reference-based haplotype assembly approaches.

### Approach

**Fig. 1:**
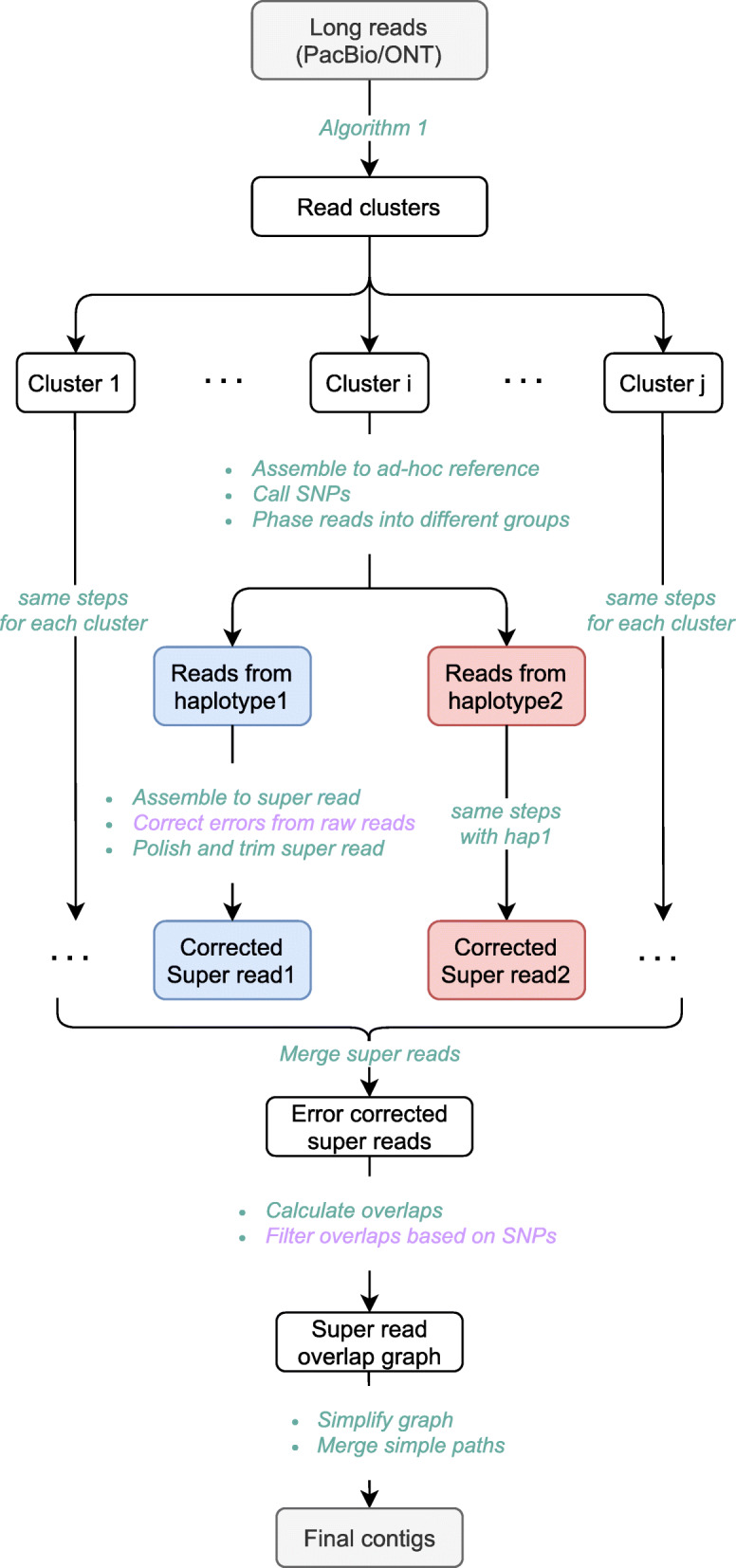
An overview of phasebook. The purple text represents that this step is optional. *Correct errors from raw reads* is recommended for long reads with high sequencing error rate such as PacBio CLR and ONT reads. *Filter overlaps based on SNPs* is recommended for small genomes or specific genomic regions such as MHCs

See Fig. 1 for an illustration of the following description. For full details on the following outline, see descriptions of the individual steps in the workflow figure in the “Methods” section.

#### Motivation and related work

From a larger perspective, phasebook pursues a *divide-and-conquer strategy:* in the *divide* stage, it collects contiguous raw reads into small local clusters. Each of the clusters then refers to a local instance of the *minimum error correction (MEC)* problem, the solution of which yields two haplotype-specific super reads. In the *conquer* stage, all haplotype-specific super reads are assembled following the overlap-layout-consensus (OLC) assembly paradigm, using an overlap graph where nodes correspond to the super reads and edges indicate that two super reads are likely to stem from identical haplotypes.

As such, phasebook reflects a combination of an approach that solves the MEC problem without reference sequence first and performs OLC-ased assembly in a haplotype-aware setting second.

The *key insight* is to first phase (and error correct) reads locally and only then perform global assembly. Local phasing is possible because local coordinate systems can be raised in a way that does not introduce biases that hamper the process. Having phased reads before assembly (and having corrected errors accordingly) then renders global assembly using OLC techniques easy in comparison with OLC-based approaches that work with unphased data.

As a whole—combining the MEC problem in a divide stage with OLC-bsaed assembly in a conquer stage—the workflow of phasebook appears to be a novelty, to the best of our judgment. The components of phasebook are inspired by a few recent approaches.

The divide stage is inspired by WhatsHap [9], as an approach that is based on the minimum error correction (MEC) framework. However, while WhatsHap achieves good performance in phasing long third-generation sequencing reads in particular, it crucially depends on the availability of high-quality reference. Here, we set the framework for solving the MEC problem when high-quality reference genome sequence is not available.

After solving the resulting local MEC instances, phasebook adopts techniques to support haplotype-aware de novo assembly based on overlap graphs, in full consistency with prior steps, in the *conquer stage*. In this, phasebook is inspired by recent, related work on short reads that focuses on overlap graph based assembly (see SAVAGE [22] or POLYTE [23], for example). Here, we adapt these techniques to dealing with long reads. For computing the necessary overlaps, we make use of Minimap2 [24], as the superior current approach to compute overlaps among long reads in sufficiently short time also for large datasets.

#### Overview of Technical Steps

Each of the two stages falls into *two basic steps*.

In the *divide* stage, phasebook *first* determines the clusters where each of the clusters reflects a collection of reads that overlap each other in terms of genomic position (executed by “Algorithm 1” in Fig. 1).

Secondly, *during divide,* phasebook solves an instance of the MEC problem for each such cluster. For this, as abovementioned, phasebook follows the algorithmic principles of WhatsHap [9, 25]. The decisive challenge for making this possible is to provide local reference sequence with which one can solve the local instances of the MEC problem. We recall that we operate in a de novo setting, such that we cannot expect reference sequence to exist in the first place. Here, we need to provide sufficiently reliable reference sequence for the small segments of the genome the clusters of reads stem from.

**Fig. 2:**
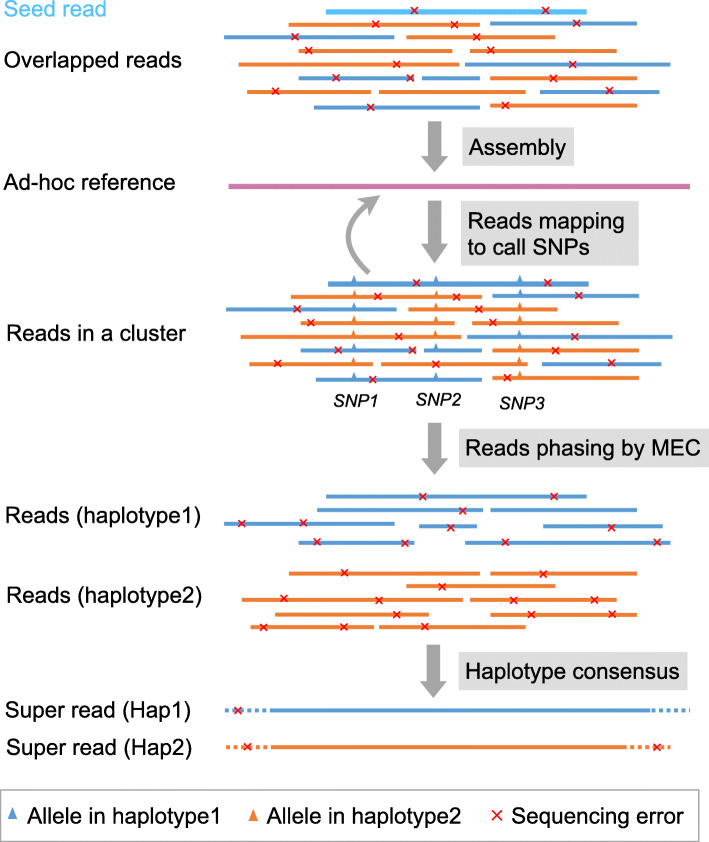
A schematic diagram for read phasing and super read generation in a read cluster. The blue reads belong to haplotype 1, and the orange reads belong to haplotype 2. The bold sky blue read means that this read is a seed read. We use WhatsHap to separate reads into two different groups based on SNPs involved in long reads. The dash line regions in super reads represent the bases with sequencing low coverage, which can be optionally trimmed to generate corrected super reads

See “Assembly” and “Reads mapping to call SNPs” in Fig. 2 for the descriptions in the following paragraph. For computing such local reference sequences for each of the read clusters, we first construct a local overlap graph, and subsequently compute an ad hoc reference for each of the clusters based on the overlap graph (see the “Read overlap graph” and “Ad hoc reference construction” sections). After polishing this ad hoc reference, we determine the polymorphic loci using Longshot [26], as a state-of-the-art tool for calling variants in long reads (see the “SNP calling” section); note that we need these variants for phasing reads by solving the MEC problem in the following. We determine a multiple alignment of all reads in a cluster based on the ad hoc reference. Overall, the procedure implies in particular to have setup reference coordinates, as provided by the positions in the ad hoc reference, that are sufficiently free of sequential biases.

In Fig. 1, this comprises all steps from “assemble to ad hoc reference” to “polish and trim super read” (where polishing and trimming may be required in low coverage areas). As pointed out above, see also Fig. 2 for further illustrations. Solving the local MEC problem for each cluster results in two super reads that reflect local haplotypes for each cluster (one of which generated as per the blue, and one of which generated as per the red branch in Fig. 1).

**Fig. 3:**
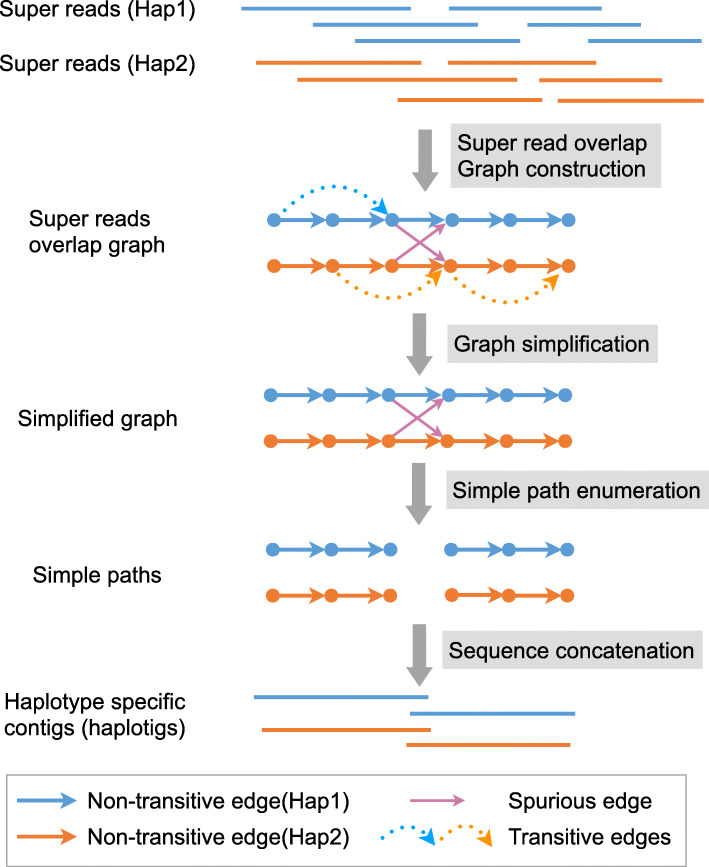
A schematic diagram for super read overlap graph construction. The blue super reads belong to haplotype 1, and the orange super reads belong to haplotype 2. The solid arrow lines (blue or orange) represent non-transitive edges, and the dash arrow lines represent transitive edges. The solid arrow lines (reddish purple) represent spurious edges caused by incorrect super read overlaps from different haplotypes

In the *conquer* stage, phasebook *first* constructs an overlap graph on all super reads emerging from the divide stage (comprising all steps from “Merge super reads” to “Filter overlaps based on SNPs” in Fig. 1). In this graph, edges indicate that two overlapping super-reads stem from the same haplotype, based on statistical considerations. The number of super reads is much smaller than the number of raw reads, which renders the construction of this overlap graph easily manageable (which arguably is a virtue of the design of the workflow overall). Because super reads are haplotype-specific, the corresponding overlap graph virtually consists of two components each of which reflects one of the haplotypes, with only a handful spurious edges connecting the components, see Fig. 3 for further illustration.

Finally, in the *second step of the conquer stage*, super reads are assembled into haplotype-specific contigs by careful elimination of redundant and spurious edges, where the identification of spurious edges is based on evaluating (putative) polymorphic sites. After edge elimination, phasebook lays out paths in the resulting connected components of the overlap graph, indicated by “Simplify graph” and “Merge simple paths” in Fig. 1.

See the “Methods” section for full details on each of the sub-steps involved in the overall workflow.

### Datasets

#### Simulated data

We simulated various datasets using different long-read sequencing technologies, namely, PacBio CLR, PacBio HiFi, and ONT. Generally, PacBio CLR and ONT reads have high sequencing error rate (5 ∼ 15%), while PacBio HiFi reads have much lower error rate (< 1*%*). We selected two human MHC haplotypes from the Vega Genome Browser: PGF and COX. Subsequently, we used PBSIM [27] model-based simulation to generate PacBio CLR reads of N50 length 20 kbp and average sequencing error rate 10% for each of the two haplotypes at a coverage of 25 ×. We used PBSIM sampling-based simulation to generate PacBio HiFi reads of N50 length 10 kbp for each of two haplotypes at a coverage of 15 × by utilizing human individual HG00731 as the sample profile, which was downloaded from EBI. Besides, we used NanoSim V2.6.0 [28] as an approved simulator to generate ONT reads of N50 length 20 kbp and average sequencing error rate 10% using the built-in pre-trained model human_NA12878_DNA_FAB49712_albacore for each of the two haplotypes at a coverage of 25 ×. For each sequencing platform, the reads from each of the two haplotypes were combined respectively, resulting a read set for a pseudo-diploid genome (PGF and COX). Additionally, we used PBSIM model-based simulation to generate PacBio CLR reads of N50 length 20 kbp and average sequencing error rate 10% for each of the two haplotypes at a coverage of 15 ×, 25 ×, 35 ×, and 45 ×, in order to evaluate the effect of sequencing coverage.

#### Real data

To evaluate our method on real sequencing data, we downloaded PacBio HiFi and CLR sequencing reads of human individual HG00733 from EBI and SRA (accession number: SRR7615963) and Oxford Nanopore PromethION sequencing reads of human individual NA19240 from ENA where the project ID is PRJEB26791. Subsequently, we extracted reads primarily aligned to chromosome 6, which is ∼ 170 Mbp in length. The approximate sequencing coverage per haplotype of chromosome 6 for these three types of long reads is 18 ×, 44 ×, and 26 ×. Full-length haplotypes have been reconstructed for HG00733 and NA19240 in a recent study [29] by applying multi-platform sequencing technologies and specialized algorithms. Hence, we downloaded the corresponding haplotype sequences as the ground truth for benchmarking experiments with the focus on chromosome 6. As for a whole human genome, we benchmarked tools on the individual HG002, on all three kinds of long reads. In addition, we benchmarked tools on PacBio CLR sequencing data of *Arabidopsis thaliana* (*A. thaliana*). More details about data sources are shown in “Availability of data and materials” section.

### Run other approaches for comparison

#### Reference-guided haplotype assembly

Haplotype assembly methods such as WhatsHap and HapCut2 take as input a reference genome sequence and a variant set (unphased VCF file), and output a phased VCF file. For simulated data, we selected another MHC haplotype (named DBB) as the reference also from the Vega Genome Browser. For real data, we extracted the sequence of chromosome 6 from GRCh38 as the reference for HG00733 (Chr6) and NA19240 (Chr6) datasets. Likewise, we used the GRCh38 and assembly TAIR10.1 as references for HG002 and *A. thaliana* datasets, respectively. Note that the Chr6 haplotypes of HG00733, NA19240, and HG002 are quite diverged from GRCh38 Chr6, and the TAIR10.1 is one of the parental strain of the sequenced *A. thaliana*. Subsequently, we aligned sequencing reads to reference genomes and called variants using Minimap2 and Longshot, respectively. Ultimately, we followed the method proposed in [23] to reconstruct contigs for each haplotype block from phased VCF files using bcftools consensus.

#### De novo haplotype aware assembly

For the sake of a fair comparison of the performance of the assembly programs, we chose to use the results which keep the haplotype information rather than collapse it. Consequently, we used the combination of primary contigs and associated contigs for Hifiasm and Falcon, and added the option –keep-haplotypes when running Flye, for haplotype-aware comparisons. For the remaining assemblers, we just compared with the contigs as output, because of the lack of alternative options. Note that we failed to run Falcon-Unzip on our datasets^1^, because no adequate read overlaps were generated likely due to too low coverage, thus only results of Falcon are reported. To avoid excessive, redundant computations for whole genomes, we downloaded the assemblies of HG002 (HiFi, ONT) whenever available as previously published. For HG002 (CLR), we successfully ran tools such as Canu, WhatsHap, and HapCut2 but failed to run others because of limited computational resources.

In addition to phasebook itself, we implemented a strategy that combines stand-alone error correction (and not necessarily combining error correction with phasing as in phasebook) with phasebook’s HiFi mode, referred to as *phasebook-hi* in the following. As for error correction, we used Canu because it offers error-corrected versions of the raw reads as convenient, separate output. The idea behind phasebook-hi was the development of a somewhat more aggressive version of phasebook that trades off exactness in terms of phasing (as demonstrated by more misassemblies and switch errors by phasebook-hi) with enhanced contiguity (as indicated by more long haplotigs in phasebook-hi). Preempting a detailed discussion, it seems that phasebook-hi leads to improvements in general when dealing with relatively variant poor diploid genomes (such as *A. thaliana*). We benchmarked phasebook-hi on both simulated and real data (PacBio CLR and ONT reads) for comparison.

### Assembly performance evaluation

The assembly performance was evaluated by means of several commonly used metrics, routinely reported by QUAST v5.1.0 [30], as a prominent tool that evaluates assemblies *relative to a reference* (see the “Metrics” section for specific explanations. For reference-guided haplotype assembly approaches, we generated contigs for each haplotype from phased variants, even if haplotypes share highly similar regions. De novo assembly approaches, however, can assemble genomic sequences from regions shared by the different haplotypes only once, which can be interpreted misleadingly. To circumvent the issue, for evaluating reference-guided methods, we used the parameter –ambiguity-usage one, whereas for de novo assemblers, we used the parameters –ambiguity-usage all and –ambiguity-score 0.999 so as to obtain reasonable alignments of contigs with both haplotypes.

In addition to QUAST, we used Merqury v1.3 [31] as a *reference-free* genome assembly evaluation tool. This way, one could assess the performance relative to the individual haplotypes, whenever parental sequencing data was available which was applicable for all genomes under consideration here (see more details in section “Commands and versions of tools used for comparison” in Additional file 1).

### Metrics

#### Haplotype completeness

Haplotype completeness is measured by two metrics, namely, haplotype coverage (HC) and *k*-mer recovery rate.

Haplotype coverage is the percentage of aligned bases in the ground truth haplotypes covered by haplotigs, which is reported by QUAST and used to measure the overall completeness of the assembly.

*k*-mer recovery rate is reported by Mercury and consists of two aspects. First, *k*-mer completeness (*k*-mer recovery “All” in Tables 1, 2, and 3) is equal to the number of solid *k*-mers found in the assembly divided by the solid *k*-mers in the read set. Second, maternal and paternal hap-mer completeness (*k*-mer recovery “Mat” and “Pat” in Tables 1, 2, and 3) is calculated analogously, but only for the haplotype it refers to.

**Table 1:** Benchmarking results for PacBio HiFi data

Dataset	Assembler	Size (Mb)	HC (%)	*k*-mer recovery(%)			Continuity (bp)			QV	Switch error(%)	*N* (%)	Dup (%)
				All	Mat	Pat	NG50	NGA50	Phased N50				
MHC (HiFi 15x)	phasebook-hi	11.2	99.4	99.9	99.9	99.5	546,476	539,378	383,554	46.7	0.04	0.0	1.19
	HiCanu	9.5	90.8	100.0	99.8	99.9	4,827,925	517,913	460,744	42.1	0.00	0.0	1.23
	Flye	5.1	56.0	96.0	77.4	78.1	138,470	10,861	58,919	44.0	2.79	0.0	0.92
	Hifiasm	9.7	97.3	100.0	99.9	100.0	4,878,334	2,205,550	620,366	59.8	0.01	0.0	1.21
	IPA	8.6	81.9	99.5	92.7	99.6	1,426,746	818,630	589,604	47.8	0.03	0.0	1.15
	Wtdbg2	4.7	49.8	91.2	47.0	70.5	–	–	72,528	38.6	3.76	0.0	0.77
	*HapCut2*	9.0	56.5	84.7	54.7	54.0	257,599	155,079	167,061	31.4	5.90	9.0	1.50
	*WhatsHap*	9.0	59.4	84.7	55.2	53.7	257,599	155,079	176,555	31.4	6.26	9.0	1.43
HG00733 (Chr6) (HiFi 18x)	phasebook-hi	378.6	91.2	99.3	96.7	96.3	517,478	491,919	317,664	47.6	2.08	0.0	1.53
	HiCanu	344.6	83.9	99.7	97.3	97.4	1,456,437	991,541	440,882	39.2	1.76	0.0	1.42
	Flye	169.0	55.1	97.9	53.3	49.3	–	–	70,128	45.0	11.40	0.0	0.97
	Hifiasm	341.5	93.5	99.7	97.1	96.5	28,008,203	10,513,146	673,184	46.4	1.81	0.0	1.12
	IPA	280.5	73.3	99.2	82.8	86.0	1,612,661	722,645	460,373	41.7	1.95	0.0	1.21
	Wtdbg2	167.3	54.8	97.3	49.4	47.3	–	–	72,850	40.6	16.09	0.0	0.97
	*HapCut2*	340.8	76.5	99.3	91.7	91.1	379,321	354,305	330,339	40.8	6.48	0.4	1.3
	*WhatsHap*	340.9	76.5	99.3	91.7	91.1	381,196	359,841	327,329	40.9	6.52	0.4	1.3
HG002(HiFi 14x)	phasebook-hi	6709	–	97.5	80.0	85.0	136,140	–	111,668	50.5	0.33	0.0	–
	HiCanu	2953	–	97.1	49.8	54.0	–	–	937,018	57.9	0.15	0.0	–
	Falcon	2955	–	97.3	49.5	62.2	–	–	501,274	49.1	0.40	0.0	–
	Hifiasm	3067	–	97.5	49.6	65.3	–	–	1,146,665	54.0	0.11	0.0	–
	*HapCut2*	6435	–	98.8	87.8	90.2	145,138,636	–	858,407	40.0	1.64	5.4	–
	*WhatsHap*	6435	–	98.8	87.8	90.2	145,138,636	–	858,156	40.0	1.64	5.4	–

The sequencing technology and the average sequencing coverage per haplotype are shown in the first column. The MHC dataset is simulated whereas the others are real. Size (Mb) represents the size of assemblies generated by each assembler. Due to lack of high-quality phased assemblies as the ground truth, haplotype coverage and NGA50 for HG002 are not provided. NGA50/NG50 calculation uses a diploid genome size (double haploid genome size). The haploid genome size of MHC, HG00733(Chr6), and HG002 is 4.7 Mb, 171 Mb, and 3.1 Gb, respectively. HC(%) is the haplotype coverage. In the *k*-mer recovery (%) multicolumn, all is the *k*-mer completeness for both haplotypes combined, mat is maternal hap-mer completeness and pat is paternal hap-mer completeness. *N* (%) is the ambiguous bases proportion. Dup (%) is the duplication ratio. The assemblers marked as italics (*HapCut2* and *WhatsHap*) are *reference-guided* methods, whereas the others are de novo assembly methods. Note that we compared with IPA in MHC and HG00733(Chr6) datasets, the official PacBio assembler for HiFi reads instead of Falcon. The publicly released assemblies of HG002 (Canu, Falcon, Hifiasm) were directly used for comparison

**Table 2:** Benchmarking results for PacBio CLR data

Dataset	Assembler	Size (Mb)	HC (%)	*k*-mer recovery(%)			Continuity (bp)			QV	Switch error(%)	*N* (%)	Dup (%)
				All	Mat	Pat	NG50	NGA50	Phased N50				
MHC (CLR 25x)	phasebook	10.8	95.2	96.7	88.0	76.3	172,577	172,577	133,141	37.7	0.66	0.0	1.36
	phasebook-hi	14.3	98.0	97.5	89.4	88.8	361,721	354,437	122,594	40.6	6.26	0.0	1.88
	Canu	5.9	59.2	96.3	80.7	76.9	2,184,005	62,395	65,217	39.4	4.57	0.0	0.92
	Falcon	5.4	60.5	94.3	82.2	68.8	4,814,264	32,719	120,818	27.6	5.24	0.0	1.17
	Flye	5.0	74.1	94.2	64.2	70.5	548,628	66,242	74,992	37.1	5.34	0.0	1.01
	Wtdbg2	4.7	58.9	90.5	46.9	63.2	–	–	102,431	33.0	5.49	0.0	0.93
	*HapCut2*	9.1	56.4	84.2	53.7	53.0	393,164	254,386	282,817	31.3	5.93	8.9	1.52
	*WhatsHap*	9.2	56.6	84.2	53.4	53.2	393,164	254,386	279,136	31.2	5.62	8.8	1.52
HG00733 (Chr6) (CLR 44x)	phasebook	453.2	92.9	98.7	89.7	90.6	256,934	253,785	164,373	32.6	5.50	0.0	1.92
	phasebook-hi	291.0	81.0	97.9	68.2	65.3	587,151	552,300	201,382	33.9	14.41	0.0	1.33
	Canu	178.0	56.8	97.9	52.7	52.9	110,328	–	119,821	38.8	17.57	0.0	0.98
	Falcon	185.6	63.4	95.1	41.8	42.0	155,444	132,950	142,167	28.6	22.22	0.0	1.04
	Flye	168.1	51.9	97.6	25.4	75.8	–	–	2,094,032	42.9	3.12	0.0	0.99
	Wtdbg2	165.2	64.6	88.9	35.3	35.3	–	–	142,300	24.2	20.07	0.0	1.0
	*HapCut2*	341.3	61.8	99.3	92.1	91.7	3,899,799	1,944,878	1,346,888	41.0	5.65	0.4	1.57
	*WhatsHap*	341.3	63.4	99.3	92.1	91.6	3,349,274	1,944,877	1,334,202	40.9	5.72	0.4	1.53
HG002 (CLR 25x)	phasebook	5829	–	92.3	60.1	59.2	96,740	–	70,473	31.9	1.61	0.0	–
	phasebook-hi	6590	–	97.1	62.0	70.6	312,775	–	150,123	35.2	7.16	0.0	–
	Canu	3119	–	97.1	49.5	62.3	47,412	–	207,853	40.0	6.49	0.0	–
	*HapCut2*	6435	–	98.8	87.9	90.2	145,138,636	–	1,756,246	40.1	1.52	5.4	–
	*WhatsHap*	6435	–	98.8	87.9	90.2	145,138,636	–	1,729,580	40.1	1.52	5.4	–
A. thaliana (CLR 75x)	phasebook	301	–	89.7	76.6	76.0	66,120	–	39,513	27.0	2.78	0.0	–
	phasebook-hi	296	–	94.9	89.5	88.8	301,078	–	149,964	33.4	4.25	0.0	–
	Canu	238	–	94.4	86.8	86.6	204,191	–	64,998	31.9	4.88	0.0	–
	Flye	142	–	87.1	62.8	62.5	35,796	–	30,209	30.0	7.54	0.0	–
	Wtdbg2	125	–	35.1	26.4	26.3	11,374	–	35,733	14.4	15.17	0.0	–
	*HapCut2*	238	–	91.3	99.4	53.3	18,585,056	–	531,267	36.4	5.73	0.2	–
	*WhatsHap*	238	–	91.3	99.5	53.3	1,8585,056	–	1,637,965	36.3	5.72	0.2	–

Due to lack of high-quality phased assemblies as the ground truth, haplotype coverage and NGA50 for HG002 and *A. thaliana* are not provided. We failed to run Falcon, Flye, and Wtdbg2 for the HG002 (CLR) data on a computing machine (48 cores, 1 TB RAM) probably because of running out of memory. The method phasebook-hi represents a combination of performing Canu’s error correction and trim module on raw noisy long reads and then performing genome assembly for corrected reads with phasebook (HiFi mode)

**Table 3:** Benchmarking results for ONT data

Dataset	Assembler	Size (Mb)	HC (%)	*k*-mer recovery(%)			Continuity (bp)			QV	Switch error(%)	*N* (%)	Dup (%)
				All	Mat	Pat	NG50	NGA50	Phased N50				
MHC (ONT 25x)	phasebook	14.5	97.0	98.4	94.0	87.7	152,599	152,599	102,954	36.9	0.78	0.0	1.82
	phasebook-hi	14.8	91.6	97.5	89.4	88.8	796,423	746,361	176,895	40.6	6.99	0.0	1.96
	Canu	5.9	59.6	95.4	83.2	65.9	1,419,330	93,217	100,918	33.4	6.44	0.0	0.84
	Falcon	6.3	67.8	94.3	82.2	68.8	2,489,921	442,832	220,087	27.6	4.65	0.0	1.10
	Flye	5.0	54.5	95.0	81.4	62.6	792,993	86,893	106,195	39.7	6.22	0.0	0.82
	Shasta	5.0	63.0	86.8	62.5	48.5	957,250	136,119	147,280	23.7	10.71	0.0	0.89
	Wtdbg2	5.0	76.5	89.1	67.0	47.4	4,821,843	208,838	136,335	24.3	4.33	0.0	0.99
	*HapCut2*	9.1	55.9	84.5	55.6	52.9	493,723	279,218	367,400	31.5	6.33	8.9	1.53
	*WhatsHap*	9.2	56.9	84.4	55.3	53.0	493,723	279,218	324,450	31.5	5.47	8.9	1.51
NA19240 (Chr6) (ONT 26x)	phasebook	380.8	84.4	87.5	53.9	55.1	90,219	85,595	51,291	20.6	36.96	0.0	1.87
	phasebook-hi	335.5	79.4	83.7	46.3	45.9	127,968	122,097	63,798	22.3	40.12	0.0	1.76
	Canu	169.2	65.4	83.9	40.7	40.8	–	–	129,586	22.5	35.40	0.0	0.98
	Falcon	179.3	64.0	77.7	33.7	35.4	69,660	37,993	162,461	20.5	35.72	0.0	1.02
	Flye	167.4	57.3	89.4	40.1	48.3	–	–	115,043	25.1	31.85	0.0	0.99
	Shasta	164.9	62.7	76.8	33.2	34.2	–	–	190,758	20.2	39.98	0.0	0.99
	Wtdbg2	166.7	60.6	78.3	33.2	33.9	–	–	160,729	20.4	35.13	0.0	0.98
	*HapCut2*	341.3	56.2	96.3	71.2	71.2	27,552,232	3,285,944	105,889	23.0	29.39	0.4	1.68
	*WhatsHap*	341.3	56.4	96.2	71.1	71.3	27,552,232	3,520,811	102,096	23.0	28.45	0.4	1.70
HG002 (ONT 38x)	phasebook	5691	–	92.0	62.2	63.2	390,659	–	202,723	26.6	2.28	0.0	–
	Canu	2901	–	84.5	39.3	51.6	–	–	266,624	23.2	10.27	0.0	–
	Flye	2928	–	83.9	38.5	50.6	–	–	287,619	23.0	11.94	0.0	–
	Shasta	2805	–	88.8	40.9	53.2	–	–	276,802	25.0	12.59	0.0	–
	Wtdbg2	2794	–	83.0	34.4	44.2	–	–	239,707	22.8	9.62	0.0	–

The publicly released assemblies of HG002 (Canu, Flye, Shasta, Wtdbg2) were directly used for comparison. We failed to run HapCut2 and WhatsHap for HG002 data on a computing machine (48 cores, 1 TB RAM) probably due to running out of memory

#### Contiguity

N50, NG50, NGA50, and Phased N50 (phased block N50) are used to measure the contiguity of an assembly. N50 is defined as the length for which the collection of all contigs of that length or longer covers at least half of the assembly. NG50 is similar to N50 except that half of the true genome, and not the assembly needs to be covered. NGA50 is similar to NG50 insofar as contigs are replaced by only the blocks of the contigs that can be consistently aligned with the true genome. Phased N50 is similar to N50 where instead of contigs, phased blocks of contigs are considered; unlike the other metrics, Phased N50 is reported by Merqury, but not by Quast.

#### Accuracy

Quality value (QV) and switch error rates, as reported by Merqury, are estimated in a reference-free way. Besides, N-rate is defined as the proportion of ambiguous bases (‘N’s) in the assembly, which is reported by QUAST.

#### Duplication ratio

Duplication ratio is equal to the total amount of aligned bases in the assembly divided by the total amount of aligned bases in the reference sequences, which is reported by QUAST.

### Benchmarking experiments

#### Summary

In the following evaluation, we focus on the criteria that relate to performance in terms of phasing, like haplotype coverage, *k*-mer recovery rate, NGA50 and Phased N50, and switch error rate, apart from a basic evaluation of haplotigs in terms of error content, first and foremost (see Tables 1, 2, and 3 for the corresponding statistics). Please note that values like N50 and NG50, and contig length in general, are relatively meaningless in a setting that addresses to assemble genomes in a haplotype-aware manner (contig length alone comes regardless of haplotype awareness, so highlights effects that can be seriously misleading). We therefore list these numbers only for completeness, see Additional file 1: Tables S1, S2 and S3 for the corresponding statistics.

We performed benchmarking experiments including all methods on the simulated and real data as described above, for all three types of long reads, namely, PacBio HiFi, PacBio CLR, and Oxford Nanopore reads (see details in the section “Commands and versions of tools used for comparison” in Additional file 1). In summary, the results show that for both simulated and real PacBio HiFi reads, Hifiasm and phasebook share top performance. phasebook apparently reconstructs more complete (indicated by HC, *k*-mer recovery), comparably equally continuous (indicated by NGA50, phased block N50), and accurate (QV, switch error, N-rate) haplotigs than the other approaches. While Hifiasm is on a par with phasebook on HiFi data, advantages of phasebook are most evident for PacBio CLR, and Nanopore reads, where it outperforms other methods in terms of haplotype coverage on both simulated and real data in terms of a relatively large margin. At the same time, phasebook maintains better or similar performance in terms of various other metrics (NGA50, phased block N50, QV, switch error, and N-rate). More details on the results are provided in the following sections.

#### PacBio HiFi reads

Table 1 shows the assembly statistics on PacBio HiFi reads. In the simulation experiment (MHC), phasebook achieves the largest haplotype coverage (HC 99.4%), while on real data (HG00733-Chr6), it reconstructs the second largest part of the haplotypes (HC 91.2%), rivaled only by Hifiasm (HC 93.5%). On whole-genome real data (HG002), phasebook also obtains the second largest the haplotype completeness (all 97.5%, maternal 80.0%, paternal 85.0%), outperformed only by the reference-guided approaches HapCut2 and WhatsHap (all 98.8%, mat 87.8%, pat 90.2%). Apart from HapCut2 and WhatsHap requiring a reference genome, this is further compensated by the fact that HapCut2 and WhatsHap fail to achieve a large fraction of the haplotype sequence when dealing with Chromosome 6 (HC, only <76.5%), the effects of which are likely to persist also on the entire genome. As for assembly contiguity, phasebook outperforms Flye and Wtdbg2 among de novo assemblers in terms of NGA50 and Phased N50 in both MHC and HG00733-Chr6 datasets.

The currently most predominant reference-guided methods (WhatsHap and HapCut2) only yield less than 60% and 77% haplotype coverage in MHC and HG00733-Chr6 datasets, respectively, though achieving the highest *k*-mer recovery in HG002. Meanwhile, they have NGA50 and phased block N50 about twice as short in MHC compared with phasebook, whereas yielding similar and longer length in HG00733-Chr6 and HG002 datasets, respectively. Further, they have lower QV and much higher switch error rates in comparison with phasebook on all three datasets, and they clearly the largest N-rates in comparison with other methods. All these effects can be attributed to making use of a reference genome. De novo assemblies, which do not suffer from biases that are inherent to reference genome based approaches, do not have such high switch errors and N-rates (in fact they do not contain any *N*).

See also Additional file 1: Table S1 for contig length statistics. While the N50 of phasebook is much smaller, the amount of very long contigs is substantially greater than that of other approaches, so proves to be very well able to compute very long contigs. In general, phasebook generates substantially more contigs, which explains the relationship between N50 and long contigs. We point out again that contig length only has ambiguous value in the context of haplotype-aware assembly. The great N50 for Flye and Wtdbg2, for example, could be due to collapsing bubbles and smoothing tangled regions in the assembly graph, which is indicated by the relatively large amount of erroneous bases and misassemblies for the two tools, which further also prevents them from separating the two phases.

#### PacBio CLR reads

See Table 2 for the following discussions. Note that here we do not only consider phasebook itself, but also its more aggressive version phasebook-hi (reflecting to error correct reads first, where here Canu’s error correction is used, and then applying phasebook’s HiFi mode to the pre-corrected reads).

In comparison with all other approaches, phasebook achieves the second largest and the largest haplotype coverage on the simulated MHC region (HC 95.2%) and on the real HG00733-Chr6 (HC 92.9%) data, respectively (HC 52∼74*%* throughout for all other approaches). phasebook-hi achieves the largest and the second largest haplotype coverage in MHC (HC 98%) and HG00733-Chr6 (HC 81%) data. Moreover, phasebook-hi outperforms Canu in terms of both maternal and paternal *k*-mer recovery. On HG002, phasebook only achieves higher maternal haplotype completeness compared with Canu. In *A. thaliana* data, phasebook shows higher haplotype completeness than other de novo assemblers such as Flye and Wtdbg2 but is outperformed by Canu in this respect. However, the best performance is achieved by phasebook-hi in *k*-mer recovery (all 94.9%, mat 89.5%, pat 88.8%), compared with all other methods. Interestingly, reference-based methods achieve high maternal, but rather low paternal *k*-mer recovery. This is very likely explained by the fact that high-quality maternal genome sequence has been used as the reference for these reference-dependent tools (note that this choice reflects the practically most reasonable decision, because the genome of the mother is the genome that resembles the genome under analysis the most and is of highest quality).

Apart from this, phasebook and phasebook-hi outperform all other de novo approaches in terms of NGA50 (MHC, HG00733-Chr6) and NG50 (HG002, A. thaliana) quite drastically. In *A. thaliana*, phasebook has shorter NG50 than Canu, while phasebook-hi has the greatest NG50. phasebook also achieves the lowest switch error rate across all methods in MHC and *A. thaliana* data, and the second lowest switch error rate in HG00733-Chr6 and HG002. On HG002, phasebook is rivaled only by the reference assisted methods in that respect. Note further that Flye, which outperforms phasebook in terms of QV (Flye 42.9; phasebook 32.6) and switch error rate (Flye 3.12; phasebook 5.50), Flye obtains truly lower haplotype coverage (Flye 52%; phasebook 92.9%), and no NGA50, which puts this into perspective.

The reference-guided methods WhatsHap and HapCut2 generate more contiguous haplotigs (larger Phased N50 and NG(A)50). Nevertheless, as we already observed in PacBio HiFi read data, this comes at the cost of substantially elevated N-rates and switch errors. Additionally, these methods only reconstruct 56∼63*%* haplotype coverage in MHC and HG00733-Chr6 datasets, which is much lower in comparison with phasebook.

phasebook-hi generally trades higher switch error rates (being on a par with those of the other methods, while phasebook itself tends to have substantially lower switch error rates)) in compensation for greater (haplotype-aware) contiguity of the assembly, so may present an interesting option for certain applications. On *A.thaliana*, phasebook-hi even appears to be the best method overall. A likely explanation is that the modified protocol of phasebook-hi is favorable on diploid genomes that are relatively variant sparse.

We also evaluated the effects of using correcting errors for raw reads (step “Correct errors from raw reads” in Fig. 1) or not, where correcting reads is the default. See Additional file 1: Tables S5 and S9: results for MHC and HG00733-Chr6 show that using this option improves the assembly in terms of contiguity and accuracy (QV, switch error), and also improves haplotype coverage on HG00733-Chr6 quite substantially (Additional file 1: Table S5), at the expense of 4 times more CPU time (Additional file 1: Table S9). The general recommendation therefore is to use it, unless one cannot afford the requested amount CPU time. Note, however, that the requested amount does not appear to be excessive, as being roughly on a par with that of other methods.

As for contig length statistics regardless of haplotype awareness, see Additional file 1: Table S2, similar effects as for HiFi data show. While showing relatively small N50, phasebook, and phasebook-hi in particular, generate larger amounts of long contigs than the other methods. This shows that phasebook’s assemblies are similarly fragmentary as those from the other methods.

#### Oxford Nanopore reads

See Table 3 for the following results. Importantly, phasebook achieves the largest haplotype coverage on all (simulated) MHC (HC 97.0%), (real) NA19240-Chr6 (HC 84.4%), and (real) HG002 (overall/maternal/paternal *k*-mer recovery, 92%/62.2%/63.2%) data compared with all other approaches (HC 56∼77*%* in MHC, 56∼65*%* in NA19240-Chr6; overall/maternal/paternal *k*-mer recovery 83∼89*%*/34.4∼40.9*%*/44.2∼51.6*%* in HG002).

Meanwhile, phasebook-hi achieves the second largest haplotype coverage in MHC and NA19240-Chr6 data. In addition, phasebook generates haplotigs with the lowest switch error rate (about 6∼14 times lower) and second largest QV (Flye having best QV) on MHC data. phasebook-hi further outperforms other de novo assemblers in terms of NGA50 on MHC and NA19240-Chr6, while phasebook outperforms alternative approaches on NA19240-Chr6 and HG002 data (evaluation of phasebook-hi on HG002 was not possible because of not being able to run Canu on HG002 by ourselves due to a lack of resources at the time of the analysis). Meanwhile, both phasebook and phasebook-hi achieve similar Phased N50 in MHC and HG002 data.

Reference-guided methods generate more contiguous haplotigs (larger N50, phased block N50, and NGA50 values). As we have already observed in PacBio HiFi/CLR datasets, this comes at the expense of significantly higher N-rate and switch errors, especially in MHC data. Notably, these methods only obtain approximately 56% haplotype coverage in MHC and NA19240-Chr6 data, which is much inferior to the result reconstructed from our reference-free approach.

As for contig length statistics, we observe effects very similar to those from HiFi and CLR reads: relatively small N50 values are put into perspective by large amounts of long contigs (in particular on the whole human genome HG002, phasebook delivers the by far greatest amount of contigs longer than 500 kb). This demonstrates that phasebook, just as the other methods, is very well possible to generate very long contigs. At any rate, we would like to recall that basic contig length statistics can be rather misleading in the evaluation of ploidy aware assemblers.

### Effect of sequencing coverage

In order to evaluate the effect of sequencing coverage on noisy long read assembly performance, we used two MHC haplotypes (PGF and COX) as a template for a diploid genome. We then simulated PacBio CLR reads with different sequencing coverage, namely, 15 ×, 25 ×, 35 ×, and 45 × per haplotype. Subsequently, we ran phasebook and all other tools on these four datasets. The benchmarking results are shown in Additional file 1: Table S4.

As the sequencing coverage varies, phasebook always achieves the best performance in terms of haplotype coverage (95∼97*%*), clearly outperforming all other tools (52∼76*%*). In addition, it yields haplotigs with much lower switch error rate (0.6∼1.3*%*, 3 to 10 times lower) at all different sequencing coverages. At a coverage of 15 ×, phasebook has the lowest QV with respect to all other methods, while being second best on all other coverage rates, with Canu taking first place in that respect (QV phasebook 37.7∼41.2 versus 39.4∼41.8 from Canu).

The explanation for this effect is that phasebook corrects sequencing errors at haplotype level, whereas other methods correct errors by considering reads from the two haplotypes jointly. This yields improvements at low coverage rates for the other methods; however, other methods do not improve error rates on increasing coverage. Note in general that correcting sequencing errors in noisy long reads experiences difficulties starting from 15 × and less. Besides, the haplotype coverage of phasebook slightly improves on increasing sequencing coverage, and the accuracy (QV and switch errors) of phasebook is improved when sequencing coverage increases from 15 × to 25 ×; furthermore, only slight improvements are observed when raising sequence coverage to 35 × and 45 ×.

### Runtime and memory usage evaluation

The runtime of phasebook is dominated by two steps. First, the computation of all-vs-all long read overlaps for which we use Minimap2, as one of the fastest approaches to compute read overlaps. Second, generating corrected super reads consumes a considerable amount of time, as a consequence of the high rate of sequencing errors in long reads.

Of note, the number of read clusters is approximately linear with the genome size, as a logical consequence of the design of the clusters. Converting massive amounts of raw reads into a much reduced set of super reads, parallelized across clusters, and only then constructing overlap graphs on (the reduced amount of) super reads, and deriving assemblies from this (comparatively small) graph greatly speeds up the process, and avoids consumption of excessive amounts of memory.

In an overall account, our approach is perfectly apt for assembling large genomes, such as human genomes or other diploid genomes of similar length.

We evaluated the runtime and memory usage of related tools on both simulated and real data for all three kinds of long read data (namely, as usual, PacBio HiFi, PacBio CLR, and Oxford Nanopore reads). Results are shown in Additional file 1: Table S6, S7, S8, respectively.

In detail, for PacBio HiFi data, Additional file 1: Table S6 shows that phasebook takes 64.8 h CPU time on Chr6 data and 6636 h CPU time on whole human genome data (HG002). In comparison with HiFiasm, the strongest competitor, phasebook requires 1.5 and 18 times more runtime, but requires only at most half of the memory on HG002. In comparison with other approaches, phasebook requires more time, while still being on the same order of magnitude for Chr6 (other de novo approaches: 14.4 to 41.0 h). In terms of peak memory usage, phasebook requires roughly similar amounts like Canu and Flye (on MHC and Chr6). As was to be expected, reference-guided methods, such as WhatsHap and HapCut2, have advantages in terms of runtime and space consumption.

For PacBio CLR reads, Additional file 1: Table S7 shows that phasebook, Canu, and Falcon are orders of magnitude slower when assembling CLR reads relative to HiFi reads. phasebook requires 1129 h CPU time on Chr6 data, which is similar to Falcon, and twice the amount of Canu. phasebook-hi instead requires even less time than Canu, while still requiring more than Flye and Wtdbg2. In addition, Additional file 1: Table S7 shows that phasebook consumes about 7213 h CPU time on the whole human genome data (HG002), which is faster than Canu (31,933 h) and also phasebook-hi (28,357 h). As shared by those methods, this indicates that the error correction routine requires substantially larger amounts of time than that of phasebook itself. Note that this logic turns around for *A. thaliana*: here, both Canu and phasebook-hi require substantially less time than phasebook, which underscores the impression that these approaches are favorable for variant poor, diploid genomes

For ONT, Additional file 1: Table S8 indicates that phasebook takes 727 h CPU time on Chr6 data, which is faster than Canu (1139 h), but somewhat slower than Falcon (508 h), with Flye, Shasta, and Wtdgb2 being the clearly least runtime intense tools. phasebook takes about 19,564 h CPU time on the whole human genome data, which reflects a normal amount of time in general.

Overall, this demonstrates that phasebook and phasebook-hi generally appear to be relatively slow in comparison with other tools, while, however, never requiring runtimes that exceed those of others by orders of magnitude. In addition, phasebook is well behaved in terms of memory usage. In summary, both phasebook versions are very well applicable in all real world scenarios of interest.

## Discussion

Haplotype-aware assembly of diploid genomes, which preserves haplotype identity of the assembled sequences, plays a crucial role in various applications. Long-read sequencing data appears to be particularly suitable for addressing this task since long reads can capture linkage of genetic variations across much longer ranges than short reads. However, the elevated error rates of long reads induce considerable difficulties, because it can be challenging to distinguish errors from true variations. The error rates are the major reason why existing long-read assemblers tend to collapse assemblies into consensus sequence, thereby loosing haplotype information. Further, the majority of existing methods for generating haplotype-aware assemblies tend to require guidance by reference genomes.

The difference in error profile characteristics of the most popular long-read technologies, such as PacBio HiFi, PacBio CLR, and Oxford Nanopore add to the challenge, because it seems that each sequencing technology requires specially tailored tools, where each tool integrates the particularities of the individual sources of errors. In summary, this explains why prior methods were struggling to address to generate haplotype-aware de novo assemblies for diploid genomes for all predominant sequencing technologies, despite the currently overwhelming interest in such tools.

We have introduced phasebook to overcome this gap in terms of existing methods: phasebook is (to the best of our knowledge) the first method that successfully addresses all of these (crucial) points. phasebook reconstructs the haplotypes of diploid genomes, supporting usage of all predominant sequencing technologies, namely PacBio HiFi, PacBio CLR, and Oxford Nanopore, de novo, that is without requiring external guidance. The results demonstrate the superiority of the new approach in terms of the majority of approved assembly related metrics.

Key to success for phasebook is to not just follow standard assembly paradigms (such as de-Bruijn or overlap graph based assemblies). Rather, phasebook adopts ideas from the original (non-de novo) WhatsHap, as a predominant tool for reference-guided read based phasing, and reframes these ideas in a reference-free setting.

In somewhat more detail, phasebook casts the overall assembly problem as several local instances of the minimum error correction (MEC) problem, and assembles the resulting solutions of the local instances thereafter. The challenge inherent to this is to establish the instances of the MEC problem correctly; while this is straightforward in a reference-aided setting, the setup of appropriate MEC instances in a de novo setting requires careful consideration of various issues.

Solving the instances corresponds to determining local haplotype-specific patches of genomics sequence. Just as for the original WhatsHap these patches are extremely accurate in terms of error content. This kills two birds with one stone: the local sequence patches do not only account for accurately preserving haplotype identity, but also for correcting errors in the original (long) reads.

The high accuracy of the resulting sequence patches, referred to as super-reads, then allows for a fairly straightforward procedure that finalizes the generation of high-quality assemblies: One raises an overlap graph on the super reads and determines the longest paths in this overlap graph. Because super-reads are (1) long, (2) accurate, and (3) haplotype-specific, the resulting long and accurate overlaps reliably identify the haplotypes also across regions of the genomes that extend the read length by considerable amounts: Full-length assemblies can be easily determined by computing longest paths in the overlap graph. To further polish assemblies, we implement options for sequencing error correction and read overlap refinement that account for differences in terms of sequencing platform characteristics.

Benchmarking results on both simulated and real data indicate that phasebook achieves the best performance in terms of haplotype coverage, often reconstructing up to 20–30% more haplotype-specific sequence than the toughest competitors. At the same time, phasebook maintains better or comparable performance in terms of various other relevant aspects. We also have presented phasebook-hi, as a more aggressive approach, which trades misassemblies for contiguity overall, by correcting reads for errors regardless of haplotype identity. This proves to be favorable for rather variant poor genomes, such as *A. thaliana*. The original approach to partition reads into haplotype-specific groups before correcting errors, instead, proves to be superior on sufficient amounts of polymorphic sites in the genome.

Our comparisons consider all state-of-the-art tools that address to assemble diploid genomes in a way that takes ploidy into account. Note that although the focus is on de novo assembly, we also consider reference-guided approaches so as to judge the current state of the art from a larger perspective, which probably indicates the superiority of partitioning reads into haplotype-specific groups before sequencing error correction.

Among the three kinds of long-read sequencing data, PacBio HiFi data is the most promising one in terms of distinguishing the different haplotypes thanks to its low error rates. PacBio CLR and Nanopore reads, on the other hand, come with elevated error rates. As usual, combining long and highly erroneous reads with accurate short reads may establish a viable alternative to achieving even better performance than phasebook, at the expense, of course, of additional efforts in terms of sequencing experiments.

In summary, the results demonstrate that phasebook successfully reconstructs individual haplotypes of the most polymorphic region (MHC) in the human genome at utmost quality in terms of completeness, contiguity, and accuracy. The integration of phasebook in population-scale long-read sequencing projects may open up novel opportunities.

Still, there is room for improvements. Most importantly, phasebook is confined to work with diploid genomes, so cannot handle polyploid genomes. We consider it worthwhile future work to expand on the idea of polyploids and to design and implement a method that can deal with polyploid genomes, or even cancer and metagenomes, while adopting the ideas of WhatsHap style approaches further.

## Conclusions

We have presented phasebook, a novel approach for reconstructing the haplotypes of diploid genomes from long reads de novo, that is without the need for a reference genome. The approach is implemented in an easy-to-use open-source tool https://github.com/phasebook/phasebook. Benchmarking results on both simulated and real data indicate that our method outperforms state-of-the-art tools (both haplotype assembly and de novo assembly approaches) in terms of haplotype coverage, while preserving competitive performance or even achieving advantages in terms of other various aspects relevant for genome assembly. Therefore, the integration of phasebook may reveal new opportunities in population-scale long read sequencing projects.

## Methods

### Read cluster generation

#### Read overlap calculation

We firstly compute all-vs-all overlaps for raw reads using Minimap2 [24], whose seed-chain-align procedure is known to perform pairwise alignments extremely fast, so can manage the large amount of read pairs we need to process. Secondly, resulting bad overlaps are filtered by reasonable, additional criteria. For example, overlaps that are too short that do not exceed a minimum level of sequence identity and that reflect self-overlaps, duplicates, or internal matches are removed. In this, we follow Algorithm 5 in [32].

#### Read clusters

We sort reads by their length in descending order, considering that longer reads tend to have more overlaps. Processing reads in the corresponding order therefore results in larger read clusters, which reduces the number of clusters, increases the length of the resulting super-reads and hence improves the assembly overall. In each iteration, the longest read having remained unprocessed is selected as the seed read. The corresponding cluster is then determined as the set of reads that overlap the seed read (according to the criteria listed above), and all of its reads are discarded from the sorted list of reads. The procedure stops when all reads have been processed. See the details in Algorithm 1.


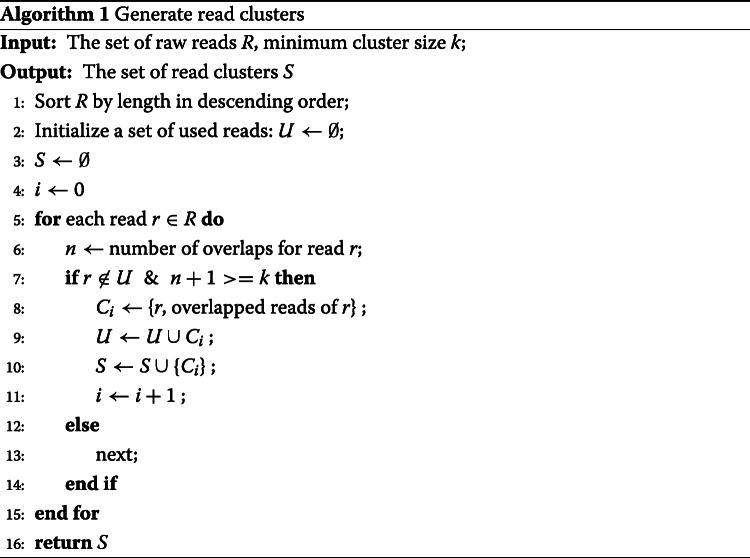


### Read phasing

Since read clusters contain reads from both haplotypes, we further phase reads into two haplotype-specific groups. See Fig. 2 for the explanation of this step.

#### Read overlap graph

For each read cluster, we largely follow the method proposed in [22, 23] to construct a read overlap graph *G*=(*V,E*) where nodes *v*∈*V* represent reads and edges *e*=(*v,w*)∈*E* indicate overlaps that are consistent in terms of length, sequence identity (see the“Parameter settings” section). Edges (*v,w*) are directed indicating that the suffix of *v* is likely to be identical with the prefix of *w*. Note that the overlaps were already determined when computing clusters. Appropriate thresholds for sequence identity and length of the overlaps are user defined.

#### Ad-hoc reference construction

In order to reduce the complexity of the overlap graph, we remove transitive edges, which reflects a common procedure. An edge *u*→*v* is called transitive if there is a vertex *w* and edges *u*→*w,w*→*v*. Subsequently, we determine the longest path in this directed acyclic graph, and the reads that give rise to it. The resulting reads are then assembled into an ad hoc reference by concatenating the overlapping reads that give rise to the path.

#### SNP calling

To remove remaining sequencing errors, and improve variant (SNP) calling and phasing, we polish the ad hoc reference using Racon [33]. The raw reads are then mapped back to the ad hoc reference. SNPs are detected by utilizing a pair-hidden Markov model, as implemented in Longshot [26].

#### Read phasing

Reads within a cluster can stem from both haplotypes. One can phase the reads conveniently, because the majority of them tend to be sufficiently long to bridge neighboring polymorphic sites. For performing the phasing, we make use of WhatsHap [25], as a tool that has been shown to operate at superior performance rates when phasing long reads. WhatsHap centers on the weighted minimum error correction problem, providing a solution with runtime linear in the number of variants. The solution corresponds to partitioning the reads in two groups, each of which immediately corresponds to one of the two local haplotypes of the diploid genome.

### Corrected super read generation

#### Super read construction

We construct a super-read for each of the two haplotype-specific groups of reads resulting from the phasing procedure. For each read group, we construct another read overlap graph, now comprising only reads as nodes that belong to the same haplotype. Using the overlap graph, we then assemble the reads in each group into a long haplotype-specific sequence, which we refer to as *super-read*. As a result, we generally obtain two super reads for each read cluster (see Fig. 2). Notably, there are two situations to consider when handling with homozygous regions since no SNP can be found. Firstly, all the reads in a cluster cannot be phased into two groups. Then, only one super-read will be computed. Secondly, some reads in a cluster can be phased into two groups, and some other reads cannot be safely assigned to phase groups. In this case, we assign the corresponding reads to both groups. Thus, they contribute to both super-read haplotypes.

#### Error correction from raw reads

This step is optional and recommended for long reads with high sequencing error rate such as PacBio CLR and Nanopore reads, while it is not necessary when processing PacBio HiFi reads because of their great accuracy. For small genomes or specific genomic regions such as MHCs, we make use of a method for self-correction of long reads in each read group. This method combines a multiple sequence alignment (MSA) strategy with local de Bruijn graphs and is implemented in CONSENT [34]. When dealing with large genomes (e.g., Chr6), to increase efficiency, we use the MSA-based error correction modules built in MECAT2 [35] and NECAT [36] for PacBio CLR and Nanopore reads, respectively. Reads in one group are supposed to stem from the same haplotype, thus one can avoid the elimination of true variation, which happens when reads from different haplotypes are mixed together. Therefore, because of the prior arrangement of partitioning reads into haplotype-specific groups, the corrected reads enjoy error rates that are lower than when running these tools on reads referring to unphased settings.

#### Super read polishing and trimming

Using the error corrected reads, we return to the super-reads computed earlier, and polish each super-read by comparing it with the now error corrected reads. (When using PacBio HiFi reads, we apply the procedure without prior correction of errors.) As a result, the super-reads come with error rates that are comparable to the ones obtained for the error-corrected raw reads. We finally trim the super read, removing parts of low coverage of raw reads. The final result is an error corrected super-read.

Note that the steps in the “Read phasing” and “Corrected super read generation” sections can be parallelized conveniently across clusters, because computations within clusters are independent of other clusters.

### Super read overlap graph construction

#### Super read overlap graph

The super read overlap graph *G*^′^=(*V*^′^,*E*^′^) is very similar to the read overlap graph, except that it is constructed from the set of corrected super reads instead of raw reads (see Fig. 3). Thus, each node *v*∈*V*^′^ corresponds to a super read, and there is an edge *v*_*i*_→*v*_*j*_∈*E*^′^ if they have sufficient overlap length and sequence identity. For the last point, consider that the super-reads exhibit very low sequencing error rates, so near-perfect identity in the overlap is a necessary condition for edges.

Because the number of super reads is substantially smaller than the number of raw reads, the super read overlap graph can be constructed in short time. For constructing it, we again compute all-vs-all super read overlaps using Minimap2, filter bad overlaps (remove self-overlaps, internal overlaps, etc.), and eventually establish the overlap graph analogous to the procedure of “Read phasing” section, where the only difference is that parameters differ; in general, because of the low error rates and the extended length of super reads, more stringent thresholds are necessary.

#### SNP-based spurious edge removal

As there may still be errors in super reads, one still needs to distinguish between uncorrected errors and true variations. As a consequence, super reads can have false-positive overlaps when considering sequence identity in the overlapping regions alone, which leads to the introduction of misassemblies.

To control false-positive overlaps, we further refine the edges of the super read overlap graph by applying a heterozygous SNP-based procedure to remove spurious edges that we developed. In more detail, for a pair of overlapping super reads, we collect the corrected reads which belong to the read groups that correspond with the super reads and map the reads back to the sequence formed by concatenating the pair of super reads along their overlap.

Subsequently, we genotype the variants involved in the overlap regions and remove overlaps that contain at least *k* heterozygous SNPs. In our experiments *k*=0, which correspond to requiring perfect overlap. This step might be time-consuming if genome size is large. We therefore consider it optional and recommend it preferably for small genomes or particular genomic regions, such as the MHC region in our case.

### Final contig generation

#### Graph simplification

First, we simplify the super read overlap graph by removing tips, because tips are likely to be introduced by spurious overlaps, hence reflect mistaken junctures of super reads. Second, we remove all transitive edges, for the exact same reasons for which we remove such edges in the read overlap graph (see the “Read phasing” section and Fig. 3).

#### Merging simple paths

A node *v*∈*V*^′^ in the directed graph *G*^′^ is defined as a *non-branching node* if its indegree and outdegree are both equal to one, and *v* is defined as a *branching node* if either its indegree or outdegree is greater than one. A simple path is a maximal non-branching path, that is its internal nodes are non-branching nodes, while starting and final nodes are branching. For such paths, there is only one possible way to combine the corresponding super reads. Therefore, we merge every simple path into a single contig. Since the number of nodes and edges in super read overlap graph *G*^′^ is small, it is straightforward and very efficient to enumerate and merge all simple paths (see Fig. 3).

#### Final contig polishing

This step is recommendable when the sequencing coverage is low, i.e., less than 20 × per haplotype. The reason is that the ends of super reads are likely to contain an elevated amount of errors. This needs to be addressed and corrected. So, for each contig generated from a simple path, we collect all corrected reads involved in the read groups that gave rise to the super reads forming the simple path and align them to the contig. By evaluation of the resulting alignment, further errors can be eliminated, resulting in polished contigs (longer than 1 kbp) as the final output.

### Parameter settings

There are mainly two parameters to be set during the read overlap graph construction, namely, the minimum overlap length and the minimum sequence identity of the read overlap. In our experiments, the minimum overlap length is 1000 bp, and the minimum sequence identity of the read overlap is 0.95 for PacBio HiFi reads, and 0.75 for PacBio CLR and ONT reads, in agreement with the approximate expected amount of identity according to the inherent error rates. These parameters reflect similar choices used with other tools [12, 14].

## Supplementary Information

**Additional file 1** Supplement: This contains all supplementary materials referenced in the main text.

**Additional file 2** Review history.

## Data Availability

The data used to generate PacBio HiFi sample profile when simulating HiFi reads can be downloaded from EBI http://ftp.1000genomes.ebi.ac.uk/vol1/ftp/data_collections/HGSVC2/working/20190925_PUR_PacBio_HiFi/[29]. For real sequencing data, PacBio HiFi/CLR reads of human individual HG00733 can be downloaded from EBI http://ftp.1000genomes.ebi.ac.uk/vol1/ftp/data_collections/HGSVC2/working/20190925_PUR_PacBio_HiFi/and SRA (accession: SRR7615963), respectively [29]. Oxford Nanopore PromethION sequencing reads of human individual NA19240 can be downloaded from ENA (project ID: PRJEB26791) [29]. PacBio HiFi, CLR, and ONT reads for HG002 can be downloaded from https://github.com/human-pangenomics/HG002_Data_Freeze_v1.0 [6, 17, 37]. PacBio CLR reads of *Arabidopsis thaliana* can be downloaded from SRA (accession: PRJNA314706) [12]. As for ground truth reference sequences used for benchmarking experiments, three human MHC haplotypes (PGF, COX, and DBB) are from the Vega Genome Browser http://vega.archive.ensembl.org/info/data/MHC_Homo_sapiens.html. Full-length haplotypes (chromosome 6) of individuals HG00733 and NA19240 can be downloaded from http://ftp.1000genomes.ebi.ac.uk/vol1/ftp/data_collections/hgsv_sv_discovery/working/20180227_IlluminaPolishedPhasedSV/[29]. Assemblies of HG002 (HiFi/ONT) generated by others can be downloaded from https://zenodo.org/record/4393631#.YSQQu9MzYWr [19] and https://s3-us-west-2.amazonaws.com/human-pangenomics/index.html?prefix=publications/SHASTA2019/assemblies/raw/[17], respectively. Simulated reads of MHCs, real reads of human Chr6 and code for reproducing results can be downloaded from Code Ocean [38]. Assemblies generated in this paper can be downloaded from Zenodo [39]. The source code of phasebook is GPL-3.0 licensed, and publicly available on Github [40].

## Footnotes

1 We contacted the authors, but received no response; note that the latest update in the GitHub repository is from 4 years ago. This seems to be in agreement with earlier attempts to contact the Falcon-unzip crew[20].
