# Supplementary material for: phasebook: haplotype-aware de novo assembly of diploid genomes from long reads
Source: Genome Biol. 2021 Oct 27;22:299. doi: 10.1186/s13059-021-02512-x (PMC8549298; doi:10.1186/s13059-021-02512-x)
Supplement: Supplementary file 1 — Additional file 1 Supplement: This contains all supplementary materials referenced in the main text. [file 13059_2021_2512_MOESM1_ESM.pdf]

Supplementary Material
phasebook: haplotype-aware *de novo* assembly of diploid genomes from long reads

Xiao Luo[1,2,†], Xiongbin Kang[1,2,†], Alexander Schönhuth[1,2,*]

[1] Centrum Wiskunde & Informatica, Amsterdam, The Netherlands
[2] Genome Data Science, Faculty of Technology, Bielefeld University, Bielefeld, Germany

[†]These authors contributed equally to the work.
[*]To whom correspondence should be addressed.

Table S1. Contig length statistics on PacBio HiFi data. #Contigs indicates the number of contigs.

**Dataset**	**Assembler**	**#Contigs**	**#Contigs** (>= 50 **Kb**)	**#Contigs** (>= 500 **Kb**)	**N50 (Kb)**	**N90 (Kb)**
MHC (HiFi 15x)	phasebook	32	31	9	501	157
	Canu	58	19	3	680	94
	Flye	17	9	4	828	292
	Hifiasm	2	2	2	4878	4799
	IPA	25	16	5	1427	165
	Wtdbg2	2	2	2	3347	1382
	HapCut2	82	42	0	258	52
	WhatsHap	82	42	0	258	52
HG00733 (Chr6) (HiFi 18x)	phasebook	1412	1380	205	450	99
	Canu	2386	639	61	1165	36
	Flye	39	29	20	16248	3368
	Hifiasm	87	38	17	28008	20361
	IPA	1050	399	60	11997	121
	Wtdbg2	32	16	9	33738	17235
	HapCut2	1886	1350	164	379	72
	WhatsHap	1886	1348	166	381	70
HG002 (HiFi 14x)	phasebook	53216	47460	310	134	91
	Canu	267	226	173	48483	8683
	Falcon	2004	987	250	33505	3001
	Hifiasm	383	185	103	98166	18089
	HapCut2	6732	674	116	145139	58618
	WhatsHap	6732	674	116	145139	58618

Table S2. Contig length statistics on PacBio CLR data.

**Dataset**	**Assembler**	**#Contigs**	**#Contigs** (>= 50 **Kb**)	**#Contigs** (>= 500 **Kb**)	**N50 (Kb)**	**N90 (Kb)**
MHC (CLR 25x)	phasebook	108	66	0	146	46
	phasebook-hi	62	59	0	299	162
	Canu	23	5	2	2184	45
	Falcon	14	4	1	4814	184
	Flye	9	7	4	935	549
	Wtdbg2	1	1	1	4726	4726
	HapCut2	38	28	4	393	155
	WhatsHap	34	28	4	393	234
HG00733 (Chr6) (CLR 44x)	phasebook	2755	2373	58	218	88
	phasebook-hi	573	560	190	724	277
	Canu	123	109	15	18252	4241
	Falcon	214	196	16	19121	881
	Flye	39	31	18	21573	3223
	Wtdbg2	279	44	26	11927	2282
	HapCut2	210	206	122	3900	910
	WhatsHap	234	228	126	3349	841
HG002 (CLR 25x)	phasebook	77672	39836	151	104	34
	phasebook-hi	30381	27566	1799	301	104
	Canu	4188	3589	466	14096	292
	HapCut2	6732	674	116	145139	58618
	WhatsHap	6732	674	116	145139	58618
A. thaliana (CLR 75x)	phasebook	6205	2115	1	56	26
	phasebook-hi	1686	1393	66	258	83
	Canu	2316	1195	80	204	42
	Flye	3027	680	29	157	24
	Wtdbg2	3533	828	0	55	17
	HapCut2	10	10	10	23460	18585
	WhatsHap	10	10	10	23460	18585

Table S3. Contig length statistics on ONT data.

**Dataset**	**Assembler**	**#Contigs**	**#Contigs** (>= 50 **Kb**)	**#Contigs** (>= 500 **Kb**)	**N50 (Kb)**	**N90 (Kb)**
MHC (ONT 25x)	phasebook	177	108	0	109	42
	phasebook-hi	40	38	11	612	165
	Canu	15	11	2	3490	95
	Falcon	15	10	2	2490	182
	Flye	7	5	4	1274	793
	Shasta	3	3	2	3860	957
	Wtdbg2	10	2	1	4822	4822
	HapCut2	40	22	6	494	234
	WhatsHap	36	24	6	494	234
NA19240 (Chr6) (ONT 26x)	phasebook	5723	3415	0	84	36
	phasebook-hi	3445	2420	9	130	48
	Canu	146	97	48	5022	1052
	Falcon	397	196	49	4764	249
	Flye	64	50	26	11457	2437
	Shasta	779	145	80	2213	513
	Wtdbg2	385	32	21	15481	2529
	HapCut2	40	32	24	27552	15696
	WhatsHap	40	32	24	27552	15696
HG002 (ONT 38x)	phasebook	30473	18766	3022	428	74
	Canu	779	677	197	33065	5992
	Flye	2031	809	208	33316	4905
	Shasta	17423	662	255	23346	3698
	Wtdbg2	5311	1199	386	15381	1343

Dataset	Assembler	Size (Mb)	Haplotype coverage(%)	k-mer recovery(%)			Continuity (bp)		QV	Switch error(%)	N (%)	Dup (%)
				All	Mat	Pat	NGA50	Phased N50				
MHC (15x)	phasebook	10.4	95.7	96.7	82.6	79.5	159789	136629	27.0	1.33	0.0	1.25
	Canu	5.6	56.4	95.0	73.6	69.9	46877	90512	35.6	5.48	0.0	1.01
	Falcon	5.5	57.1	94.2	67.6	71.0	50216	101936	31.5	5.46	0.0	0.96
	Flye	5.0	53.6	94.3	69.8	66.5	9724	75778	35.6	4.48	0.0	0.91
	Wtdbg2	4.7	52.4	90.0	40.3	62.1	-	102169	31.1	4.59	0.0	0.94
	HapCut2	9.1	56.3	84.2	53.5	53.2	254386	279136	31.2	5.29	8.9	1.52
	WhatsHap	9.2	56.5	84.2	52.9	53.4	254386	239601	31.1	5.47	8.8	1.52
MHC (25x)	phasebook	10.8	95.2	96.7	88.0	76.3	172577	133141	37.7	0.66	0.0	1.36
	Canu	5.9	59.2	96.3	80.7	76.9	62395	65217	39.4	4.57	0.0	0.92
	Falcon	5.4	60.5	94.3	82.2	68.8	32719	120818	27.6	5.24	0.0	1.17
	Flye	5.0	74.1	94.2	64.2	70.5	66242	74992	37.1	5.34	0.0	1.01
	Wtdbg2	4.7	58.9	90.5	46.9	63.2	-	102431	33.0	5.49	0.0	0.93
	HapCut2	9.1	56.4	84.2	53.7	53.0	254386	282817	31.3	5.93	8.9	1.52
	WhatsHap	9.2	56.6	84.2	53.4	53.2	254386	279136	31.2	5.62	8.8	1.52
MHC (35x)	phasebook	12.3	97.2	98.6	94.1	89.7	93110	74779	38.7	0.79	0.0	1.53
	Canu	5.8	66.9	96.3	79.5	78.0	293779	101163	41.1	4.39	0.0	0.91
	Falcon	5.6	64.5	94.8	73.1	70.5	120422	92301	32.4	5.53	0.0	1.00
	Flye	5.0	53.2	93.9	68.5	63.8	19559	67205	37.6	4.91	0.0	0.81
	Wtdbg2	4.8	70.0	91.6	48.6	61.3	-	113890	31.4	4.81	0.0	0.96
	HapCut2	9.1	56.4	84.3	54.6	53.0	254386	282806	31.3	5.53	8.9	1.52
	WhatsHap	9.2	56.6	84.3	54.6	53.0	254386	284756	31.2	5.29	8.9	1.52
MHC (45x)	phasebook	12.0	96.0	99.2	96.3	94.4	88122	75294	41.2	0.62	0.0	1.53
	Canu	6.1	60.7	96.9	82.1	83.1	120333	94921	41.8	3.26	0.0	0.94
	Falcon	5.7	59.3	95.2	73.3	74.8	93028	175191	32.5	5.34	0.0	0.85
	Flye	4.9	61.7	93.7	60.0	69.4	22165	95682	37.2	6.28	0.0	0.96
	Wtdbg2	4.8	51.6	91.3	48.0	61.7	-	134956	32.8	5.16	0.0	0.90
	HapCut2	9.2	56.4	84.3	53.7	53.4	254386	279136	31.2	5.71	8.9	1.52
	WhatsHap	9.2	57.4	84.3	53.3	53.9	254385	278832	31.2	5.48	8.8	1.50

Table S4. Benchmarking results for PacBio CLR reads with different sequencing coverage. The two MHC sequences (pseduo-diploid) were used to simulate long reads with sequencing coverage per haplotype 15x, 25x, 35x and 45x, respectively.

Dataset	Correct reads	Size (Mb)	Haplotype coverage(%)	k-mer recovery(%)			Continuity (bp)		QV	Switch error(%)	N (%)	Dup (%)
				All	Mat	Pat	NGA50	Phased N50				
MHC (CLR 25x)	yes	10.8	95.2	96.7	88.0	76.3	172577	133141	37.7	0.66	0.0	1.36
	no	12.3	96.3	97.7	87.6	87.9	92188	71443	36.9	0.90	0.0	1.64
HG00733 (Chr6) (CLR 44x)	yes	453.2	92.9	98.7	89.7	90.6	253785	164373	32.6	5.50	0.0	1.92
	no	327.3	84.8	97.3	70.8	71.8	221246	129731	30.0	12.26	0.0	1.59

Table S5. Benchmarking results for running phasebook whether using the option 'Correct errors for raw reads' or not.

Dataset	Assembler	CPU time (h)	peak memory usage (GB)
MHC	phasebook	1.1	4.1
(HiFi 15x)	Canu	0.4	4.2
	Flye	1.3	2.4
	Hifiasm	0.7	16.2
	IPA	0.4	0.6
	Wtdbg2	0.3	0.5
	HapCut2	0.4	0.9
	WhatsHap	0.1	0.8
HG00733	phasebook	64.8	27.3
(Chr6)	Canu	14.8	29.9
(HiFi 18x)	Flye	41.0	33.2
	Hifiasm	41.6	18.9
	IPA	14.4	16.9
	Wtdbg2	22.8	8.1
	HapCut2	2.9	6.0
	WhatsHap	2.2	6.0
HG002	phasebook	6636.6	85.4
(HiFi 14x)	Hifiasm	357.3	142.9
	HapCut2	116.1	23.7
	Whatshap	41.2	23.7

Table S6. Runtime and memory usage for PacBio HiFi reads. The running time of Hifiasm is from their work (Cheng *et al.* 2021). The running time of reference-guided methods (HapCut2, WhatsHap) sums up the time of all steps such as read alignment, variant calling and phasing, and contig generation.

Dataset	Assembler	CPU time (h)	peak memory usage (GB)
MHC	phasebook	15.7	35.2
(CLR 25x)	phasebook-hi	3.2	4.9
	Canu	10.2	6.1
	Falcon	3.9	10.4
	Flye	1.7	5.6
	Wtdbg2	0.6	0.6
	HapCut2	0.5	1.8
	WhatsHap	0.2	1.8
HG00733	phasebook	1129.2	41.8
(Chr6)	phasebook-hi	546.9	35.5
(CLR 44x)	Canu	681.7	27.4
	Falcon	1006.0	23.6
	Flye	103.7	46.9
	Wtdbg2	22.7	13.5
	HapCut2	7.8	7.6
	WhatsHap	7.6	7.7
HG002	phasebook	7212.7	73.2
(CLR 25x)	phasebook-hi	28356.9	79.4
	Canu	31933.0	79.4
	HapCut2	179.5	32.7
	WhatsHap	116.2	32.7
A. thaliana	phasebook	1261.9	37.5
(CLR 75x)	phasebook-hi	434.6	63.6
	Canu	489.9	13.4
	Flye	11.9	76.8
	Wtdbg2	8.3	27.7
	HapCut2	14.1	11.4
	WhatsHap	6.0	11.4

Table S7. Runtime and memory usage for PacBio CLR reads. Note that the runtime of phasebook-hi is the total time of Canu's error correction & trim step plus running phasebook with HiFi mode.

Dataset	Assembler	CPU time (h)	peak memory usage (GB)
MHC	phasebook	48.4	5.2
(ONT 25x)	phasebook-hi	3.2	5.5
	Canu	51.0	6.4
	Falcon	6.3	10.1
	Flye	1.0	5.5
	Shasta	0.2	1.1
	Wtdbg2	0.3	0.8
	HapCut2	0.6	1.7
	WhatsHap	0.2	1.6
NA19240	phasebook	726.6	47.1
(Chr6)	phasebook-hi	770.6	26.1
(ONT 26x)	Canu	1099.0	12.2
	Falcon	507.6	23.6
	Flye	66.1	45.5
	Shasta	4.7	29.9
	Wtdbg2	25.5	11.4
	HapCut2	165.2	23.4
	WhatsHap	12.5	23.4
HG002	phasebook	19564.4	78.8
(ONT 38x)			

Table S8. Runtime and memory usage for ONT reads.

Dataset	Correct reads	CPU time (h)	peak memory usage (GB)
MHC	yes	22.0	35.3
(CLR 25x)	no	5.9	34.6
HG00733	yes	1129.2	41.8
(Chr6)	no	270.6	32.8
(CLR 44x)			

Table S9. Runtime and memory usage for running phasebook whether using the option 'Correct errors for raw reads' or not.

Assemblers	HiFi	CLR	ONT
phasebook	0.0000	0.0000	0.0003
phasebook-hi	0.0000	0.0000	0.0000
Flye	0.0002	0.0003	0.0005
Hifiasm	0.0003	-	-
Wtdbg2	0.0007	0.0096	0.0172
WhatsHap	0.0015	0.0001	0.0001
HapCut2	0.0016	0.0001	0.0001
IPA	0.0137	-	-
Falcon	-	0.0001	0.0031
Canu	0.0177	0.0001	0.0009
Shasta	-	-	0.0087

Table S10. The proportion of short contigs (< 20Kb) in the assemblies. The proportion is equal to the total length of short contigs divided by the total length of all contigs in the assembly. Here, we report the statistics of Chr6.

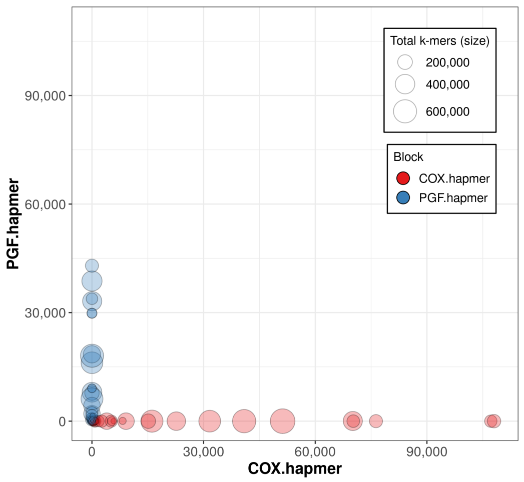

(a) MHC (HiFi)

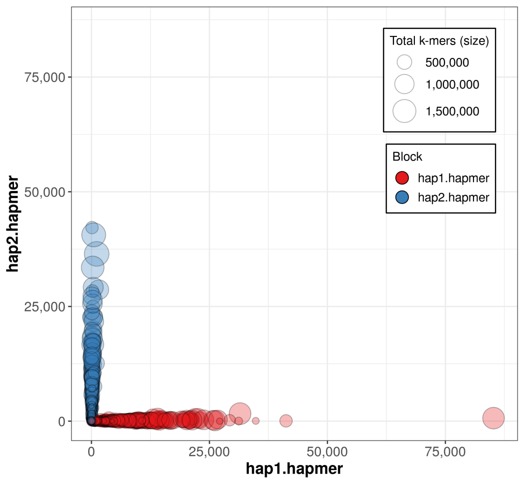

(b) HG00733:Chr6 (HiFi)

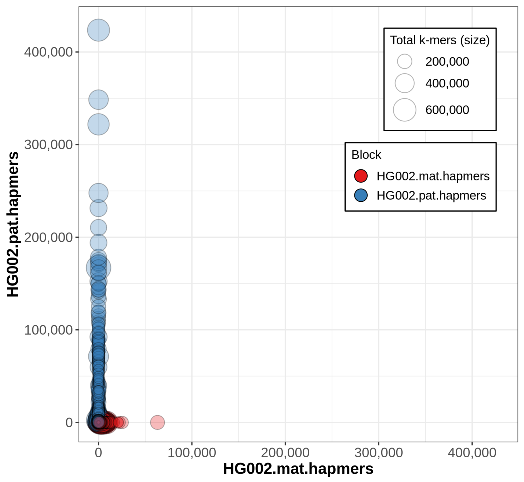

(c) HG002 (HiFi)

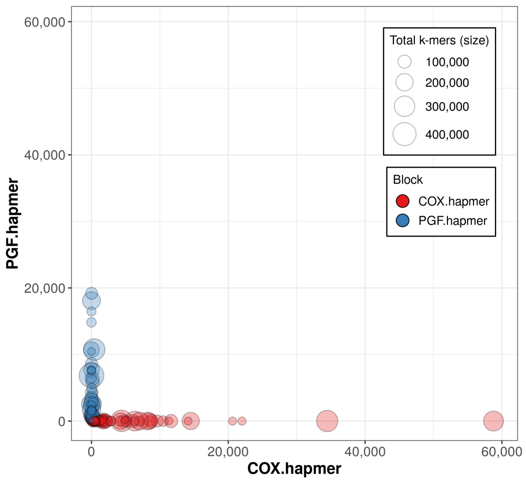

(d) MHC (CLR)

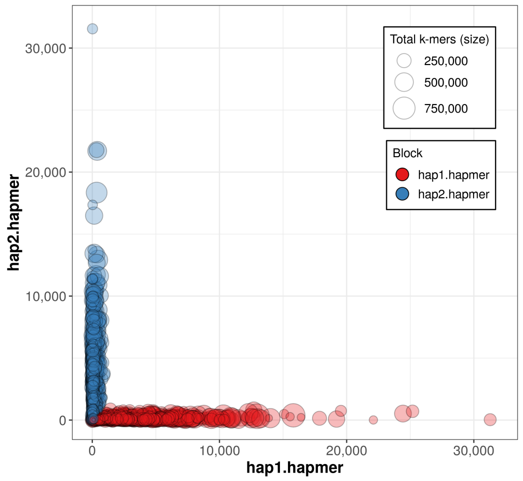

(e) HG00733:Chr6 (CLR)

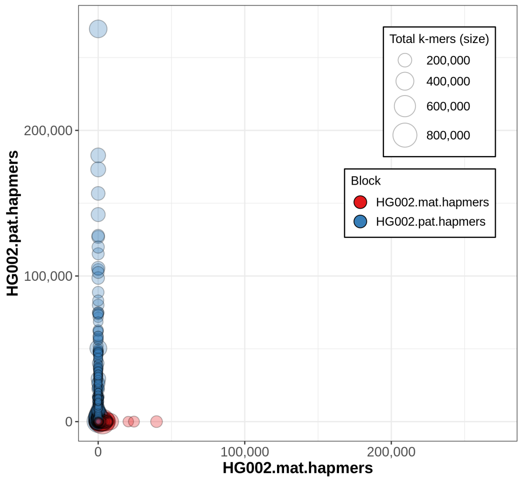

(f) HG002 (CLR)

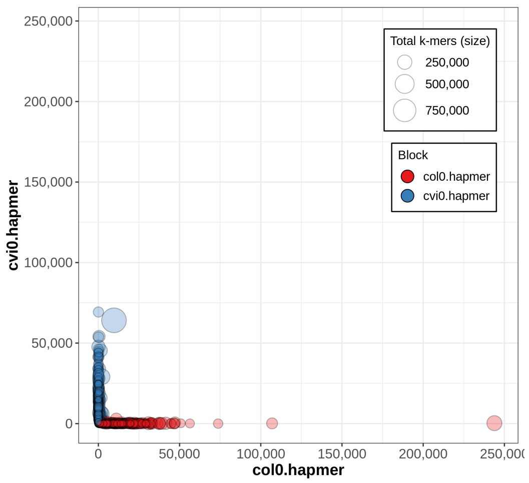

(g) A. thaliana (CLR)

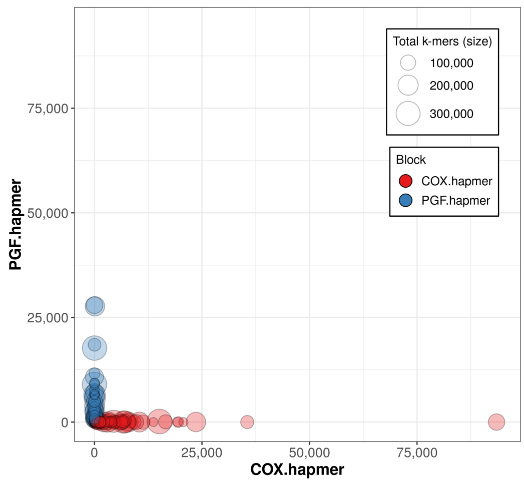

(h) MHC (ONT)

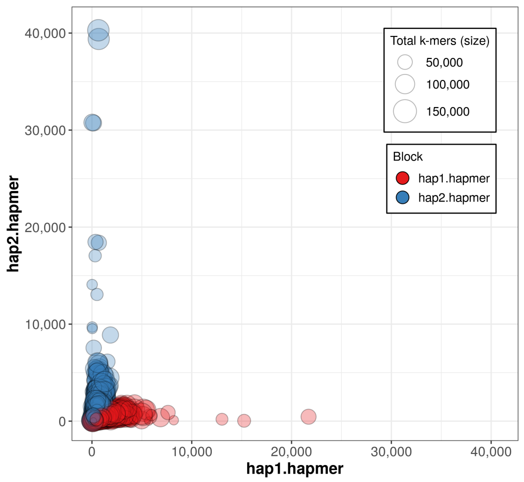

(i) NA19240:Chr6 (ONT)

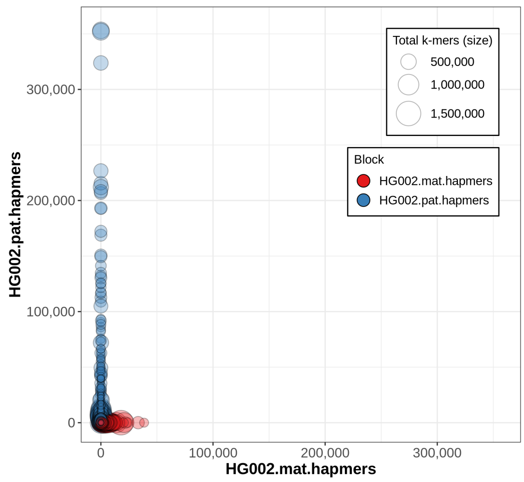

(j) HG002 (ONT)

Figure S1. Hap-mer blob plot for assemblies generated with phasebook in Table 1-3. Each circle represents a contig and its size is relative to the contig length. The color indicates maternal or paternal. The x or y axis represents the number of hap-mers found.

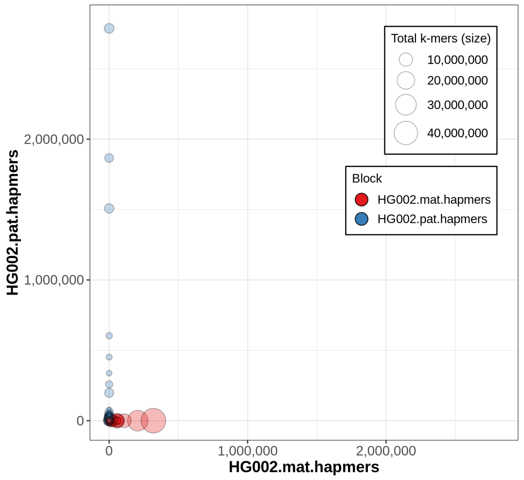

(a) HG002 (HiFi)

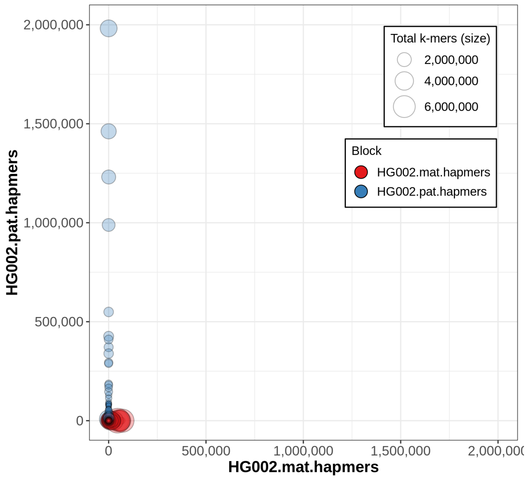

(b) HG002 (CLR)

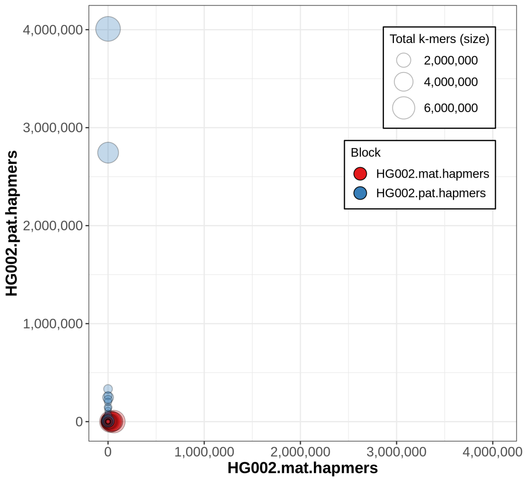

(c) HG002 (ONT)

Figure S2. Hap-mer blob plot for HG002 assemblies generated with Canu in Table 1-3.

Commands and versions of tools used for comparison

- phasebook v1.0
  Reproducible commands used to run phasebook on each dataset are available at Code Ocean: `https://doi.org/10.24433/CO.6031956.v2`. See the script `/code/reproduce.sh` for the details.

- Canu v2.1.1
  ```
  Canu -p $prefix genomeSize=$genomesize -pacbio-hifi $fq
  Canu -p $prefix genomeSize=$genomesize -pacbio $fq
  Canu -p $prefix genomeSize=$genomesize -nanopore $fq
  ```

- Falcon v1.3.0
  ```
  fc_run falcon.cfg
  ```

- Flye v2.8.2-b1689
  ```
  Flye --pacbio-hifi/--pacbio-raw/--nano-raw $fq --keep-haplotypes
  --genome-size=$genomesize --iterations 2
  ```

- Wtdbg2 v2.5-h8b12597
  ```
  Wtdbg2 -x css/rs/ont -g $genomesize -i $fq -fo out
  wtpoa-cns -i out.ctg.lay.gz -fo out.ctg.fa
  ```

- Hifiasm v0.15.4-r343
  ```
  hifiasm -o $prefix $fq
  ```

- IPA v1.3.1
  ```
  ipa local -i $fq
  ```

- Shasta v0.1.0
  ```
  Shasta --input $fq --output out
  ```

- WhatsHap v0.18
  ```
  WhatsHap phase -o $phased_vcf --reference $ref --ignore-read-groups $vcf $bam
  ```

- HapCut2 v1.3.2
  ```
  HAPCUT2 --ea 1 --fragments fragment.out --VCF $vcf --output haplotype.out
  ```

- QUAST v5.1.0rc1
  We set `--min-contig 20000`, but it has negligible effects on the results (see Table S10).
  - For MHCs
    ```
    quast.py -r $ref --ambiguity-usage all --ambiguity-score 0.999 --min-contig 20000 $fa
    ```
  - For Chr6
    ```
    quast-lg.py -r $ref --ambiguity-usage all --ambiguity-score 0.999 --min-contig 20000 $fa
    ```
  - For reference-guided methods (HapCut2, WhatsHap)
    ```
    quast.py -r $ref --ambiguity-usage one --ambiguity-score 0.999 --min-contig 20000 $fa
    ```

- Merqury v1.3
  For evaluating *A. thaliana* and HG002, we used the pre-built meryl dbs which are available at `https://obj.umiacs.umd.edu/marbl_publications/merqury/index.html`. Whereas for MHC and Chr6, we simulated Illumina reads for both haplotypes since the ground truth is known, and then prepared meryl dbs using code:

  ```
  # 1. Build meryl dbs
  for each read$i.fastq.gz
  do
      meryl k=21 count output read$i.meryl read$i.fastq.gz
  done
  ```

```
# 2. Merge
meryl union-sum output $genome.meryl read*.meryl
```

Then run Merqury for evaluation:

```
merqury.sh $readdb $mat $pat assembly.fa
```

Simulate Illumina reads:

```
art_illumina -ss HS25 -p -sam -i $ref_name.fa -f 50 -o reads.$ref_name --errfree
-l 150 -m 200 -s 10 -qs 10 -qs2 10
```
