# Supplementary material for: phasebook: haplotype-aware de novo assembly of diploid genomes from long reads
Source: Genome Biol. 2021 Oct 27;22:299. doi: 10.1186/s13059-021-02512-x (PMC8549298; doi:10.1186/s13059-021-02512-x)
Supplement: Supplementary file 2 — Additional file 2 Review history. [file 13059_2021_2512_MOESM2_ESM.docx]

Review History

**First round of review**

**Reviewer 1**

**Were you able to assess all statistics in the manuscript, including the appropriateness of statistical tests used?**

Yes: There is no stat analysis in publication.

**Were you able to directly test the methods?**

No

**Comments to author:**

The phasebook manuscript describes the new algorithm for de novo genome assembly and phasing using long read alone. The manuscript is well written with clear depictions of algorithms, definitions of technical terms, and benchmarks against other tools in this field. The core structure of the algorithm that takes into account diploid phasing for PacBio HiFi, PacBio CLR, and ONT is particularly beneficial and timely fashion with the trend in genome assembly. With its high haplotype coverage in comparison with results from other tools, I think phasebook would be beneficial for the community.

Here are my suggestions that I think would benefit the readers and users:

1. The benchmark in this manuscript is limited to only the human genome. The difference in heterozygosity and repeat contents among species would highly affect the assembled genome results of tools with particular choices of designs and parameters. While I was reading the abstract and introduction, I was under the impression that the conclusion of high haplotype coverage of phasebook came from a wide range of tested genomes. I think it worth mentioning early on that the test was done only in the human genome and only chromosome 6.

2. On page 2, the first sentence of the third new paragraph that starts with "In conclusion" should use the word "diploid" rather than "ploidy". This is because the current phasebook also handles only the diploid genome.

3. Could you add a note to clarify whether you use the trim and error correction option in phasebook for benchmarking? If you did not use them, I'm a bit surprised since the manuscript recommends this option for PacBio CLR and ONT data. (Optional) If there is additional data that shows resource usage and quality improvement from this option, it would be very useful for the scientific community.

4. Since most of the tools in this benchmarking are actively improved, it would be useful to include version numbers or git commits for each tool in the supplement.

5. For benchmarking, I think it makes the most sense to use contig rather than unitig. Even though the unitigs usually have higher accuracy, they are not fully benefit from the whole suite of algorithm and heuristic of each tool. For some tools, the N50 of contig is a few orders of magnitude higher than those of unitig. In addition, in almost all use cases, the user would use only contigs, so the benchmark with unitig would be very different from real world experience. Could you discuss the benchmark against contig instead? You may keep the results on unitig as well if you think it is useful.

6. Could you clarify how you treat the reads that could not be unambiguously assigned to one of the super-read haplotypes? There are regions in the human genome with very low heterozygosity. Are those reads duplicated and assigned to both super-read haplotypes?

7. Does the Error Rate include the mismatch due to the incorrectly assigned haplotype? Or does it contain only Error Rate after comparing substring of the assembled genome to the corresponding haplotype? If it is the former, it would be a composite of both the mis-phasing and the fine-scale error due to sequencing error. This is worth mentioning because the method to further improve the results from one of the scenarios are different.

8. (Optional) You may consider removing the section "Workflow: Paradigms and Stages", and take some of the content to combine with "Workflow: Overview of Technical Steps". Having three sections "Workflow: Sketch and Motivation; Workflow: Paradigms and Stages; Workflow: Overview of Technical Steps" are a bit excessive and redundant.

Arkarachai Fungtammasan

**Reviewer 2**

**Were you able to assess all statistics in the manuscript, including the appropriateness of statistical tests used?**

Yes: As the authors present a haplotype-aware assembly method, I highly recommend to use a trio-based phasing assessment, as described in Merqury.

**Were you able to directly test the methods?**

Yes

**Comments to author:**

The authors present Phasebook, a de novo assembler designed for more accurate and complete haplotype reconstruction. The authors demonstrate their tool on the MHC region and Chromosome 6, using simulated and real reads from PacBio HiFi, CLR, and ONT data.

The manuscript is very well written, in a thorough way in overall. However, there are a few points to be addressed to convince the phasing accuracy and its practical usage.

1. The MHC locus is one of the most hypervariable region, as the authors addressed. The high variability is actually favored for most de novo assemblers, as it is easier to distinguish each haplotype as they are so diverse. As a result, a lot of the de novo assemblers output both haplotype sequences into separate contigs. The utmost challenge is to make each haplotype sequence more continuous, without introducing any haplotype switches (especially long-range switches). The high similarity within the MHC locus is a big challenge, and a lot of the assembly graphs built from noisy long reads have difficulties overcoming this unless it was built really long reads or highly accurate reads.

To make the benchmark a fair comparison, the authors should add a comparison to contigs. None of the production level assembly projects use unitigs, as their purpose is to represent the smallest unit in the assembly graph. Most of the assemblers the authors benchmarked upon produce pseudo-haplotype contigs, and their associated contigs, or contigs that was not used to build the main contig. All of these contigs are locally intact for its haplotype, especially in the most recent HiFi based assemblies.

2. How does Phasebook perform on a whole human genome? I would encourage to run Phasebook on their tested HG00733 and NA129240. Or, better consider using HG002 from https://github.com/human-pangenomics/HG002_Data_Freeze_v1.0, which the HiFi (25kb library) and ONT data are relatively more recently obtained compared to what the authors used (SRR7615963 and PRJEB26791). Adding the HG002 whole genome assembly experiment will have the advantage of reflecting the more realistic outcomes of each sequencing data types.

3. All HG00733, NA19240, and HG002 are from a trio, which the parental Illumina data sets are available. Haplotype completeness, phase block statistics and its accuracy can be assessed with Merqury on any trio assembly. Most of the assemblers the authors benchmarked have already a published assembly of HG002, which could be directly used for comparison.

4. In addition to the phase block statistics, I would like the authors to measure the falsely duplicated sequence rate and possible collapses that Phasebook produces. Extra sequence content, duplicated from an already assembled haplotig would not be detected from the metrics the authors used in their benchmark. Likewise, the unassembled portion is unclear from the HC. Were both haplotypes not assembled? Or just one haplotype being unassembled? Consider evaluating the metrics for each haplotype separately.

**Authors Response**

**Point-by-point responses to the reviewers’ comments:**

We thank the reviewers for their insightful comments. As we feel, they have been very helpful to improve the manuscript, clarify misunderstandings and remove inconsistencies. In the following, we address all comments raised point by point.

Reviewer #1:

Comment: “The phasebook manuscript describes the new algorithm for de novo genome assembly and phasing using long read alone. The manuscript is well written with clear depictions of algorithms, definitions of technical terms, and benchmarks against other tools in this field. The core structure of the algorithm that takes into account diploid phasing for PacBio HiFi, PacBio CLR, and ONT is particularly beneficial and timely fashion with the trend in genome assembly. With its high haplotype coverage in comparison with results from other tools, I think phasebook would be beneficial for the community.”

*Response: Dear Arkarachai, we thank you for your insightful comments and are pleased with your generally positive assessment. Please see below for how we have addressed your comments.*

Comment: “Here are my suggestions that I think would benefit the readers and users:

1. The benchmark in this manuscript is limited to only the human genome. The difference in heterozygosity and repeat contents among species would highly affect the assembled genome results of tools with particular choices of designs and parameters. While I was reading the abstract and introduction, I was under the impression that the conclusion of high haplotype coverage of phasebook came from a wide range of tested genomes. I think it worth mentioning early on that the test was done only in the human genome and only chromosome 6.”

*Response: We agree that the statement was too generalizing. At the same time, we had indeed been planning to have phasebook as a tool that is able to assemble diploid genomes of various kinds.*

*Therefore, we have extended the experiments. Now, we have benchmarked on human MHC, chromosome 6, whole human genome, and, most importantly to agree with the general statement, Arabidopsis thaliana as well. See Tables 1-3: while results on the additional genomes vary and show effects that had not become evident on MHC and Chr6 alone, they agree on the usual trends in general: phasebook has excellent haplotype coverage and little switch errors.*

*Although the effects are not entirely clear because of the lack of an applicable reference, results for PacBio CLR data for Arabidopsis did not appear to outperform other approaches. Canu in particular seems to do a great job here.*

*Arabidopsis is rather variant-sparse. To appropriately account for variant poorer genomes, we developed a modified, more aggressive approach, called ‘phasebook-hi’. For this, we employ a standard error correction procedure (here adopting the one from Canu) that, unlike the original phasebook, does not take haplotype identity into account during the initial error correction stage.*

*Subsequently, we run phasebook in HiFi mode on the standard error corrected data.*

*As a result, phasebook-hi clearly outperforms all other approaches in nearly all categories both on the full human genome and Arabidopsis. The only expense is a sacrifice in terms of switch error rate, which is then roughly on a par with the alternative approaches (which is considerably better for the original phasebook).*

*In an overall assessment, this seems to demonstrate that phasebook-hi may be a useful alternative for diploid genomes that tend to be poorer in terms of variants. The essential advantages of phasebook, such as haplotype coverage and so on, get documented in both versions.*

*We have modified all corresponding paragraphs in the Results section to reflect this.*

Comment: “2. On page 2, the first sentence of the third new paragraph that starts with "In conclusion" should use the word "diploid" rather than "ploidy". This is because the current phasebook also handles only the diploid genome.”

*Response: We reworded the paragraph to reflect this appropriately.*

Comment: “3. Could you add a note to clarify whether you use the trim and error correction option in phasebook for benchmarking? If you did not use them, I'm a bit surprised since the manuscript recommends this option for PacBio CLR and ONT data. (Optional) If there is additional data that shows resource usage and quality improvement from this option, it would be very useful for the scientific community.”

*Response: During benchmarking, we have used the option ‘Correct errors for raw reads’ [see Figure1] for PacBio CLR and ONT data, but do not use this option for PacBio HiFi data because the following step ‘Polish and trim super read’ [see Figure1] is already sufficient to achieve the best performance without consuming additional time.*

*We have added additional experiments on MHC(CLR reads) and HG00733(Chr6, CLR reads) also without the option ‘Correct errors for raw reads’ to highlight any potential differences of interest. Table S5 collects the corresponding results in terms of assembly performance, and Table S9 in terms of computational resource requirements.*

*The results are described in the manuscript as well; see the second last paragraph in ‘PacBio CLR reads’ for the corresponding discussion: using the option improves on all contiguity, accuracy and haplotype coverage, while requiring 4 times runtime.*

Comment: “4. Since most of the tools in this benchmarking are actively improved, it would be useful to include version numbers or git commits for each tool in the supplement.”

*Response: Versions and commands of tools used in the benchmarking experiments have been included in the Supplement.*

Comment: “5. For benchmarking, I think it makes the most sense to use contig rather than unitig. Even though the unitigs usually have higher accuracy, they are not fully benefit from the whole suite of algorithm and heuristic of each tool. For some tools, the N50 of contig is a few orders of magnitude higher than those of unitig. In addition, in almost all use cases, the user would use only contigs, so the benchmark with unitig would be very different from real world experience. Could you discuss the benchmark against contig instead? You may keep the results on unitig as well if you think it is useful.”

*Response: Thanks for pointing this out. While we were interested to see statistics on unitigs, we agree that putting this in the focus can be misleading.*

*Now we use ‘contigs’ rather than ‘unitigs’ generated by other tools for comparison, as suggested. Now, we have removed unitigs results from the manuscript entirely, to avoid any confusion. Note as well that some most recent versions of tools, such as the most recent version of Canu, in the meantime do no longer output ‘unitigs’ file anyway.*

*Also, according to further suggestions on additional criteria, we now display more fine grained results on contig length in the Supplement, while focusing on evaluating the correctness in terms of correctly phased contig length (NGA50 and Phased N50), as suggested. Note that a table capturing all numbers would have been too large. Here, we focus on criteria reflecting performance relative to phasing, because this is the key argument of this manuscript.*

*[And, as usual, N50 alone, as so often, does not seem to point out what is really going on here as well. So we present N50 along with more detailed statistics on contig length (which point out that phasebook’s contigs are not at all shorter in general than the contigs of other approaches), to avoid inconclusive impressions.]*

Comment: “6. Could you clarify how you treat the reads that could not be unambiguously assigned to one of the super-read haplotypes? There are regions in the human genome with very low heterozygosity. Are those reads duplicated and assigned to both super-read haplotypes?”

*Response: This is a great question. There are two situations:*

*(1) All the reads in a cluster cannot be phased into two groups. Then only one super-read will be computed.*

*(2) Some reads in a cluster can be phased into two groups, and some other reads cannot be safely assigned to phase groups. In this case, we assign the corresponding reads to both groups (So, they contribute to both super-read haplotypes.)*

Comment: “7. Does the Error Rate include the mismatch due to the incorrectly assigned haplotype? Or does it contain only Error Rate after comparing substring of the assembled genome to the corresponding haplotype? If it is the former, it would be a composite of both the mis-phasing and the fine-scale error due to sequencing error. This is worth mentioning because the method to further improve the results from one of the scenarios are different.”

*Response: Thanks for this very helpful comment. We agree that it is very helpful to be able to distinguish between the two sources of errors. Here, Error Rate was referring to the former: we aligned the assembled haplotigs to the ground truth, and counted the non-matched positions (mismatches, indels) as errors. So the mismatches can be due to incorrectly assigned variations or sequencing errors (or combinations thereof).*

*To distinguish between these phenomena, we now use two metrics “quality value (QV)” and “switch error rate” so as to replace the previous metric Error Rate. See the Tables in the manuscript for the update.*

Comment: “8. (Optional) You may consider removing the section "Workflow: Paradigms and Stages", and take some of the content to combine with "Workflow: Overview of Technical Steps". Having three sections "Workflow: Sketch and Motivation; Workflow: Paradigms and Stages; Workflow: Overview of Technical Steps" are a bit excessive and redundant.”

*Response: Thanks for pointing this out, we agree that we may have been repeating ourselves. At the same time, we still were not yet pointing out clearly enough what we wanted to convey.*

*We have amended this by now having only two subsections ‘Motivation and Related Work’ and ‘Overview of Technical Steps’ in the section ‘Approach’, and having removed redundancies as much as possible. We still spend some space on explaining the approach. The intention is to convey the intuition (and the novelty) of the approach in sufficient detail for anyone who may be interested in comparing approaches from an outer perspective.*

Reviewer #2

Comment: “The authors present Phasebook, a de novo assembler designed for more accurate and complete haplotype reconstruction. The authors demonstrate their tool on the MHC region and Chromosome 6, using simulated and real reads from PacBio HiFi, CLR, and ONT data.

The manuscript is very well written, in a thorough way in overall.”

*Response: We thank the Reviewer for the generally positive assessment and the insightful comments.*

Comment: “However, there are a few points to be addressed to convince the phasing accuracy and its practical usage.

1. The MHC locus is one of the most hypervariable region, as the authors addressed. The high variability is actually favored for most de novo assemblers, as it is easier to distinguish each haplotype as they are so diverse. As a result, a lot of the de novo assemblers output both haplotype sequences into separate contigs. The utmost challenge is to make each haplotype sequence more continuous, without introducing any haplotype switches (especially long-range switches). The high similarity within the MHC locus is a big challenge, and a lot of the assembly graphs built from noisy long reads have difficulties overcoming this unless it was built really long reads or highly accurate reads.

To make the benchmark a fair comparison, the authors should add a comparison to contigs. None of the production level assembly projects use unitigs, as their purpose is to represent the smallest unit in the assembly graph. Most of the assemblers the authors benchmarked upon produce pseudo-haplotype contigs, and their associated contigs, or contigs that was not used to build the main contig. All of these contigs are locally intact for its haplotype, especially in the most recent HiFi based assemblies.”

*Response: Thanks for the detailed explanations and for pointing this out. Please note that the comment agrees with comment 5 of Reviewer 1 in several ways.*

*Statistics on unitigs primarily reflected our own interest in exploring the pros and cons of other approaches; we agree that putting numbers on unitigs in the central focus is rather misleading.*

*Also because most recent versions of some tools (such as Canu) do no longer support unitig output, we have removed statistics on unitigs from the manuscript entirely, and now focus statistics on contigs.*

*Now we use ‘contigs’ rather than ‘unitigs’ generated by other tools for comparison, as suggested. Now, we have removed unitigs results from the manuscript entirely, to not introduce confusion.*

Comment: “2. How does Phasebook perform on a whole human genome? I would encourage to run Phasebook on their tested HG00733 and NA129240. Or, better consider using HG002 from https://github.com/human-pangenomics/HG002_Data_Freeze_v1.0, which the HiFi (25kb library) and ONT data are relatively more recently obtained compared to what the authors used (SRR7615963 and PRJEB26791). Adding the HG002 whole genome assembly experiment will have the advantage of reflecting the more realistic outcomes of each sequencing data types.”

*Response: Thank you very much for reminding us to do this; we had refrained from whole genome analyses just because of the computational expenses, and arranging for the corresponding resources.*

*As suggested, we now have added benchmarking results on the whole human genome (HG002) for all three types of long reads, PacBio HiFi, CLR, and ONT reads (see Table 1-3). While results exhibit some novel effects, the general statements persist. phasebook prevails on whole genome data in terms of haplotype coverage, switch error rates and various other criteria, just as on MHC and Chr6. So, regardless of some variation in terms of results, phasebook evidently holds its promises also on whole human genome data.*

Comment: “3. All HG00733, NA19240, and HG002 are from a trio, which the parental Illumina data sets are available. Haplotype completeness, phase block statistics and its accuracy can be assessed with Merqury on any trio assembly. Most of the assemblers the authors benchmarked have already a published assembly of HG002, which could be directly used for comparison.”

*Response: Thank you very much for pointing out this nice alternative assessment tool; we had been overlooking this so far.*

*We have added various additional metrics that seemed obviously helpful to assess performance rates of interest in our setting, which puts phasing in a particular focus. In detail, we now consider also k-mer completeness, maternal/paternal hap-mer completeness, phased block N50, quality value (QV) and switch error rate, all of which are reported by Merqury. Quite evidently, this sheds valuable additional light on the relevant advantages and disadvantages of the tools.*

*We also downloaded the assemblies of HG002, as already publicly available for PacBio HiFi, ONT datasets, for benchmarking tests as suggested (see “Availability of data and materials” for the details).*

Comment: “4. In addition to the phase block statistics, I would like the authors to measure the falsely duplicated sequence rate and possible collapses that Phasebook produces. Extra sequence content, duplicated from an already assembled haplotig would not be detected from the metrics the authors used in their benchmark. Likewise, the unassembled portion is unclear from the HC. Were both haplotypes not assembled? Or just one haplotype being unassembled? Consider evaluating the metrics for each haplotype separately.”

*Response: We have added an additional metric “duplication ratio” to evaluate the extra sequence content. Duplication ratio is equal to the total number of aligned bases in the assembly divided by the total number of aligned bases in the reference sequences, which is reported by QUAST. One sees that the duplication rate of phasebook is generally greater than that of other tools, while not being excessive. Also other criteria point out that phasebook is rather conservative in joining contigs insofar as it does not join overlapping contigs if considerable doubts with respect to the introduction of switch errors remain.*

*We have evaluated the completeness for each haplotype separately. To be specific, we added the maternal and paternal hap-mer completeness reported by Merqury, see Tables 1-3 for the updated results.*

**Second round of review**

**Reviewer 1**

The researchers have addressed all the comments/concerns, improved the algorithm for HiFi use cases, and performed benchmarks on larger datasets with more comprehensive matrices. The current manuscript should be appropriate for publication.

My final recommendation is to put a pointer to "Commands and versions of tools used for comparison" in the main text when you show the benchmark.

**Reviewer 2**

I appreciate the authors for adding more detailed method description, benchmark results from HG002, phasing statistics from Merqury, and memory/runtime footprints. This greatly helped better understand the performance of phasebook. However, I found additional points that needs to be addressed, given the phasing statistics of the benchmarks, along with few minor corrections that could be made for clarity.

Please rephrase jargons used in the abstract. Be more precise about the “disturbing biases”. What biases are those? In addition, be more precise about what other aspects relevant for genome assembly is achieved.

In the background, the sentence “Overall, short-read genome assembly tools are not directly applicable to long-read data. This explains the need for novel approaches.” reads out of context. The paragraph is discussing the higher error rate in long reads but the above sentence suddenly switches the focus to short-read genome assembly tools which were never mentioned, and misses the link to the motivation of Phasebook.

Page 2. Similarly long haplotigs: perhaps “comparably long haplotigs” could be used, however reading from the tables it seems it is the case only for phase blocks. Haplotigs are contigs originating from the same haplotype.

Consider replacing all “ploidy-aware” to “haplotype-aware”. The implementation in Phasebook assumes diploid, and does not work on polyploidy genomes. Using “ploidy-aware” is misleading.

Fig. 2 indicates a seed read being used for read-to-read alignments, and an ad-hoc reference being constructed and used for calling SNVs and phasing them. The description in the main text (page 3) is rather confusing: “For computing such local reference sequences for each of the read clusters, we first determine polymorphic loci using Longshot [26], as a state-of-the-art tool for calling variants in long reads.”. How are the polymorphic loci called from a read-to-read alignment? I believe this was done on the seed read. Longshot requires a ‘reference’ and ‘aligned reads’. Make a clear distinction and clarify what reference was used for calling variants in long reads in this step.

Later, the paragraph describes “When all polymorphic loci have been determined, and a multiple alignment of all reads in a clusters has been determined accordingly, deriving reference coordinates that are sufficiently free of sequential biases is easily possible.”. Besides the grammatic error “in a clusters”, please clarify how the reference coordinates are used. Is this the ad-hoc reference? Are the polymorphic sites (their coordinates) used for the phasing using MEC? A more detail should be provided why the reference coordinates are derived. Fig. 2 has a nice schematic diagram, which could be cited more often to point which step is described in this sentence.

Page 4. Remove “reflecting a sound way”. This is unnecessary and vague expression.

The MHC haplotypes of HG00733, NA19240, and HG002 are quite diverged from GRCh38 Chr. 6. The TAIR10.1 is one of the parental strain of the sequenced A. thaliana. Putting this information upfront in the “the reference-guided haplotype assembly” section would greatly improve readers to follow the results and understand why one haplotype of the A. thaliana strain had better completeness.

On page 5, authors claim “Contigs with length less than 20kb were filtered before evaluation.” Is this limited to evaluation using QUAST? Or for all evaluation? If contigs less than 20kb are considered junk, I would encourage the authors to make it default in Phasebook not to report contigs shorter than 20kb. Reporting these small contigs is often problematic in downstream scaffolding and annotation processes, and often leads to increase in false duplication rate. Otherwise, please justify why the contigs less than 20kb were excluded from evaluation and clarify in which evaluation process it was excluded.

On page 5, and thereafter, the authors claim “contig length alone comes regardless of haplotype awareness, so highlights effects that can be seriously misleading”. I disagree with this statement and argue a good haplotype-aware assembler should generate more contiguous haplotigs. Thus, contig NG* and phase block NG* should be evaluated together, with a better assembler showing phase block NG* (and NGA*) closer to the contig NG*. I’d suggest to add contig NG50s to Table 1-3 for ease of comparison, and replace phase N50 to phase NG50.

In Table 1, specify what haploid genome size was used in each case.

In Table 1, is the phasebook benchmark done on phasebook-hi? If so, specify in the table. If the phasebook-hi was missing, add this for a fair comparison as the phasebook-hi was developed specifically for HiFi reads.

Why does the HiFi mode benchmark (phasebook-hi) on CLR and ONT assemblies have higher switch error rate and duplication rate? The reads were coming from the same haplotype, and haplotype specific variants were tracked. Not sure why suddenly this information gets lost and increases switch error rate and duplication rate. The authors could selectively apply corrections from the Canu error correction module, in order to preserve haplotype specific variants.

In Fig. 1, “Correct errors for raw reads”: I believe this should be “Correct errors from raw reads”?

How are the runs of homozygosity (ROH) region handled? I expect no variants being called resulting in no phased reads, however a super read is still generate-able. Adding these not-phase-able regions to at least one of the pseudo-haplotype would greatly increase the contig continuity by adding the missing links. Had the authors experiment with this? A discussion might be worth to add, I couldn’t find how Phasebook was handling regions when no polymorphic variant was found.

What is the output? A bag of contigs from both haplotypes? The algorithm itself is aware of the haplotype specific super-reads, in a pair-wise manner. Loosing this information seems rather odd. Is the pair-wise information tracked? This information would be very useful for further scaffolding and phasing using external sequencing platforms, such as Hi-C. Provide details of the output in the discussion.

Provide the bubble-plots from Merqury in the main figure or supplementary figure. This will visually represent the hamming error rate within a contig, and proof the contigs are haplotigs. The more phased and constructed to ‘haplotigs’, the bubbles (contigs) are expected to show up closer to the hap-mer axis. Table 1-3 are showing statistics on the back of contigs, which is good to highlight haplotype completeness, however does not provide any information on long-range switches (ie. maternal phase block to paternal phase block). If the bubbles appear close to the 1:1 diagonal, it should be clearly stated that phasebook produces pseudo-haplotype assemblies with locally accurate phase blocks. If the bubbles are appearing close to the axis, this could be highlighted better along with the description of the output.

The manuscript is missing the command lines used for the phasebook and phasebook-hi runs in the Supplements. Description and command lines used for evaluation (QUAST, Merqury) is also missing.

**Authors Response**

**Point-by-point responses to the reviewers’ comments:**

We thank the reviewers and the editor for their insightful comments. As we feel, they have been very helpful to improve the manuscript, clarify misunderstandings and remove inconsistencies. We have taken care of all comments raised by the editor. In the following, we address all comments raised by the reviewers point by point.

Reviewer #1:

Comment: The researchers have addressed all the comments/concerns, improved the algorithm for HiFi use cases, and performed benchmarks on larger datasets with more comprehensive matrices. The current manuscript should be appropriate for publication.

My final recommendation is to put a pointer to "Commands and versions of tools used for comparison" in the main text when you show the benchmark.

Response: We have done this as suggested. We added (see details in the section "Commands and versions of tools used for comparison" in the Additional file 1) in the second paragraph of subsection “Benchmarking experiments”.

Reviewer #2:

Comment: I appreciate the authors for adding more detailed method description, benchmark results from HG002, phasing statistics from Merqury, and memory/runtime footprints. This greatly helped better understand the performance of phasebook. However, I found additional points that needs to be addressed, given the phasing statistics of the benchmarks, along with few minor corrections that could be made for clarity.

Comment: Please rephrase jargons used in the abstract. Be more precise about the “disturbing biases”. What biases are those? In addition, be more precise about what other aspects relevant for genome assembly is achieved.

*Response: Done. Thanks for pointing this out.*

Comment: In the background, the sentence “Overall, short-read genome assembly tools are not directly applicable to long-read data. This explains the need for novel approaches.” reads out of context. The paragraph is discussing the higher error rate in long reads but the above sentence suddenly switches the focus to short-read genome assembly tools which were never mentioned, and misses the link to the motivation of Phasebook.

*Response: Thanks for noticing the inconsistency. We have amended this accordingly.*

Comment: Page 2. Similarly long haplotigs: perhaps “comparably long haplotigs” could be used, however reading from the tables it seems it is the case only for phase blocks. Haplotigs are contigs originating from the same haplotype.

*Response: Thanks, we have corrected this. Note that full contig length statistics, as provided in the Supplement, support ‘comparably long haplotigs’, despite that coarse measures such as ‘NG50’ make this look different.*

Comment: Consider replacing all “ploidy-aware” to “haplotype-aware”. The implementation in Phasebook assumes diploid, and does not work on polyploidy genomes. Using “ploidy-aware” is misleading.

*Response: We have replaced all “ploidy-aware” to “haplotype-aware” as suggested.*

Comment: Fig. 2 indicates a seed read being used for read-to-read alignments, and an ad-hoc reference being constructed and used for calling SNVs and phasing them. The description in the main text (page 3) is rather confusing: “For computing such local reference sequences for each of the read clusters, we first determine polymorphic loci using Longshot [26], as a state-of-the-art tool for calling variants in long reads.”. How are the polymorphic loci called from a read-to-read alignment? I believe this was done on the seed read. Longshot requires a ‘reference’ and ‘aligned reads’. Make a clear distinction and clarify what reference was used for calling variants in long reads in this step.

*Response: Thank you---indeed there was some serious confusion here. We have reformulated everything. Now formulations are consistent with algorithmic procedures.*

Comment: Later, the paragraph describes “When all polymorphic loci have been determined, and a multiple alignment of all reads in a clusters has been determined accordingly, deriving reference coordinates that are sufficiently free of sequential biases is easily possible.”. Besides the grammatic error “in a clusters”, please clarify how the reference coordinates are used. Is this the ad-hoc reference? Are the polymorphic sites (their coordinates) used for the phasing using MEC? A more detail should be provided why the reference coordinates are derived. Fig. 2 has a nice schematic diagram, which could be cited more often to point which step is described in this sentence.

*Response: See above; now formulations are in agreement with procedures.*

Comment: Page 4. Remove “reflecting a sound way”. This is unnecessary and vague expression.

*Response: Removed.*

Comment: The MHC haplotypes of HG00733, NA19240, and HG002 are quite diverged from GRCh38 Chr. 6. The TAIR10.1 is one of the parental strain of the sequenced A. thaliana. Putting this information upfront in the “the reference-guided haplotype assembly” section would greatly improve readers to follow the results and understand why one haplotype of the A. thaliana strain had better completeness.

*Response: We have added this as suggested.*

Comment: On page 5, authors claim “Contigs with length less than 20kb were filtered before evaluation.” Is this limited to evaluation using QUAST? Or for all evaluation? If contigs less than 20kb are considered junk, I would encourage the authors to make it default in Phasebook not to report contigs shorter than 20kb. Reporting these small contigs is often problematic in downstream scaffolding and annotation processes, and often leads to increase in false duplication rate. Otherwise, please justify why the contigs less than 20kb were excluded from evaluation and clarify in which evaluation process it was excluded.

*Response: We removed the sentence “Contigs with length less than 20kb were filtered before evaluation.”, which indeed tends to be misleading. We used the parameter ‘--min-contig 20000’ when running QUAST, which applies for all tools. The motivation for this threshold is that read length N50 is about 20kb, which, we believe, justifies the choice. When running Merqury, we did not filter contigs. Note that this does not induce any biases across methods.*

*For phasebook, we only output contigs of at least 1kb in length (see the end of subsection “Final contig generation” in the manuscript). Interestingly, we did not find contigs of length <20kb generated for the three Chr6 datasets (HiFi, CLR, ONT), see Table S10 in the supplement. Thus, running QUAST with or without ‘--min-contig 20000’ barely makes a difference.*

*To document all of this, we added an extra Table S10 to show the proportion of short contigs (<20Kb) in the assemblies generated by all methods. The results show that the proportion of short contigs (<20Kb) is very low for all assemblers / assemblies, which indicates using ‘--min-contig 20000’ when running QUAST has negligible effects on the results.*

*We have added extra explanations and detailed commands of QUAST and Merqury in the supplement.*

Comment: On page 5, and thereafter, the authors claim “contig length alone comes regardless of haplotype awareness, so highlights effects that can be seriously misleading”. I disagree with this statement and argue a good haplotype-aware assembler should generate more contiguous haplotigs. Thus, contig NG* and phase block NG* should be evaluated together, with a better assembler showing phase block NG* (and NGA*) closer to the contig NG*. I’d suggest to add contig NG50s to Table 1-3 for ease of comparison, and replace phase N50 to phase NG50.

*Response: We have added NG50 to Table 1-3 as suggested. Replacing phased block N50 to phased block NG50 is a good suggestion. However, Merqury only reports phased block N50 but not provides phased block NG50. (We consider implementing ‘phased block NG50’ worthwhile possible future work)*

*We agree that a good haplotype-aware assembler should generate more contiguous haplotigs. However, with the above sentence, we were intending to express that contig length statistics potentially come regardless of haplotype awareness. Imagine a haplotype-unaware assembler generating assemblies of great contiguity. Such a haplotype-unaware assembler would look great when comparing it with haplotype-aware assemblers in terms of contig length. While repeats alone prevent consensus assemblers from increasing contiguity, haplotype-aware assemblers have to deal with both repeats and ‘variant deserts’ (runs of homozygosity). This means that haplotype-aware assemblers have principled disadvantages in terms of contig length because of the runs of homozygosity that are unique in terms of sequence content otherwise.*

Comment: In Table 1, specify what haploid genome size was used in each case.

*Response: We have added this as suggested.*

Comment: In Table 1, is the phasebook benchmark done on phasebook-hi? If so, specify in the table. If the phasebook-hi was missing, add this for a fair comparison as the phasebook-hi was developed specifically for HiFi reads.

*Response: In Table 1 (HiFi reads), the benchmark is done for ‘phasebook-hi’. We have now specified this in the table.*

Comment: Why does the HiFi mode benchmark (phasebook-hi) on CLR and ONT assemblies have higher switch error rate and duplication rate? The reads were coming from the same haplotype, and haplotype specific variants were tracked. Not sure why suddenly this information gets lost and increases switch error rate and duplication rate. The authors could selectively apply corrections from the Canu error correction module, in order to preserve haplotype specific variants.

*Response: The possible reason for the higher switch error rate is that we run the Canu error correction module, which, unlike phasebook, performs error correction using reads from both of the haplotypes. Obviously, this way Canu may loose haplotype-specific variants, which phasebook, which partitions reads into haplotype sub-groups before correcting errors, does not. (Note that we claim that haplotype-aware error correction may be a decisive and so far unique achievement of phasebook.)*

*phasebook-hi on CLR and ONT tends to to generate longer super-reads having potentially longer overlaps, which translates into more duplicated sequence eventually. Some longer super-reads cannot be extended better because of implying higher switch error rate. This then leads to higher duplication rate in the final assemblies.*

*Seemingly, Canu does not provide additional options to perserve haplotype specific variants unless using TrioCanu. However, here, only single genomes are considered. (extending phasebook to related individuals and comparing it with TrioCanu is certainly interesting further research)*

Comment: In Fig. 1, “Correct errors for raw reads”: I believe this should be “Correct errors from raw reads”?

*Response: We have changed this as suggested.*

Comment: How are the runs of homozygosity (ROH) region handled? I expect no variants being called resulting in no phased reads, however a super read is still generate-able. Adding these not-phase-able regions to at least one of the pseudo-haplotype would greatly increase the contig continuity by adding the missing links. Had the authors experiment with this? A discussion might be worth to add, I couldn’t find how Phasebook was handling regions when no polymorphic variant was found.

*Response: This is a great question. There are two situations:*

*(1) All the reads in a cluster cannot be phased into two groups. Then only one super-read will be computed, such as within homozygosity (ROH) regions.*

*(2) Some reads in a cluster can be phased into two groups, and some other reads cannot be safely assigned to phase groups. In this case, we assign the corresponding reads to both groups (So, they contribute to both super-read haplotypes.)*

*We have added this explanation to the subsection “Corrected super read generation” in the Method section.*

Comment: What is the output? A bag of contigs from both haplotypes? The algorithm itself is aware of the haplotype specific super-reads, in a pair-wise manner. Loosing this information seems rather odd. Is the pair-wise information tracked? This information would be very useful for further scaffolding and phasing using external sequencing platforms, such as Hi-C. Provide details of the output in the discussion.

*Response: Thank you for this valuable comment.*

*Yes, currently, the output is a bag of contigs from both haplotypes. For super-reads themselves, we do have kept the pair-wise information being tracked in the intermediate super-read file. But for the final output, we are working on this to make pair-wise information be trackable in the final assemblies as well. Instead of discussing this in the Discussion, we describe this in the “TODO” list in the GitHub documentation. We will implement this as soon as possible, anticipating to be done before the paper is in print, and provide all necessary information in the GitHub repository.*

Comment: Provide the bubble-plots from Merqury in the main figure or supplementary figure. This will visually represent the hamming error rate within a contig, and proof the contigs are haplotigs. The more phased and constructed to ‘haplotigs’, the bubbles (contigs) are expected to show up closer to the hap-mer axis. Table 1-3 are showing statistics on the back of contigs, which is good to highlight haplotype completeness, however does not provide any information on long-range switches (ie. maternal phase block to paternal phase block). If the bubbles appear close to the 1:1 diagonal, it should be clearly stated that phasebook produces pseudo-haplotype assemblies with locally accurate phase blocks. If the bubbles are appearing close to the axis, this could be highlighted better along with the description of the output.

*Response: We have added bubble-plots of phasebook (Figure S1) and Canu (Figure S2) to the supplement as suggested. The results show that bubbles are appearing close to the axis as expected.*

Comment: The manuscript is missing the command lines used for the phasebook and phasebook-hi runs in the Supplements. Description and command lines used for evaluation (QUAST, Merqury) is also missing.

*Response: We have provided this information in the Supplement “Commands and versions of tools used for comparison”.*
